# The Involvement of Endoplasmic Reticulum Stress during the Interaction between Calcium Oxalate Crystals and Renal Tubular Epithelial Cells

**DOI:** 10.3390/biology13100774

**Published:** 2024-09-27

**Authors:** Sen-Yuan Hong, Lin-Tao Miao, Bao-Long Qin

**Affiliations:** Department of Urology, Tongji Hospital, Tongji Medical College, Huazhong University of Science and Technology, Wuhan 430030, China

**Keywords:** calcium oxalate stones, endoplasmic reticulum stress, unfolded protein response, RNA-seq

## Abstract

**Simple Summary:**

This study investigates how calcium oxalate crystals interact with renal tubular epithelial cells and contribute to kidney stone formation. By using transcriptome sequencing, the study identifies 629 differentially expressed genes when renal tubular epithelial cells are exposed to calcium oxalate crystals, with a significant number of these genes linked to endoplasmic reticulum stress and unfolded protein response. The study also highlights the potential crucial role of two specific genes, CHAC1 and FGF21, which were found to be significantly up-regulated in both cell models and kidney tissues from patients with calcium oxalate stones. The study suggests that targeting endoplasmic reticulum stress-related molecules and pathways could offer new therapeutic strategies to prevent stone formation and recurrence.

**Abstract:**

Our study aimed to elucidate the mechanisms behind the interaction between calcium oxalate (CaOx) crystals and renal tubular epithelial cells through transcriptome sequencing analysis. HK-2 cells were stimulated with or without CaOx monohydrate crystals and subjected to RNA-seq to assess the effects of CaOx crystals on gene expression changes, key pathways, and molecular players during this interaction. A total of 629 differentially expressed genes (DEGs) were identified between the control group and experimental group, with 491 genes up-regulated and 138 down-regulated. Functional enrichment analysis indicated that the DEGs were significantly associated with endoplasmic reticulum stress (ERS) and unfolded protein response. To validate our findings, we compared our results with the public dataset GSE73680 and confirmed the increased expression of two ERS-related DEGs, CHAC1 and FGF21, in renal papillary tissues from patients with CaOx stones. Collectively, these findings suggest that ERS plays a crucial role in the crystal–cell interaction and highlight the potential for developing therapeutic strategies aimed at reducing CaOx stone formation by targeting ERS-related molecules and pathways.

## 1. Introduction

Kidney stones, or nephrolithiasis, are hard deposits formed by minerals and salts within the kidneys, with calcium oxalate (CaOx) stones being the most prevalent type [1]. CaOx stones develop when calcium and oxalate concentrations in the urine reach levels that promote crystallization and aggregation. CaOx crystals can adhere to renal tubular epithelial cells (RTECs) and be internalized by the cells, and this process is referred to as the interaction between CaOx crystals and RTECs [2].

The crystal–cell interaction is thought to be a key factor in kidney stone formation, driving both initial injury and the progression of the disease. This interaction triggers pathological processes, such as oxidative stress, inflammatory responses, and cellular injury and damage. Damaged RTECs and the release of inflammatory mediators create a microenvironment conducive to further crystal adhesion, retention, and, ultimately, stone formation [3,4]. Therefore, understanding the mechanisms underlying the crystal–cell interaction is critical for developing new therapeutic strategies to prevent and treat CaOx stones. Targeting key pathophysiological phenomena involved in this interaction could offer novel approaches for reducing stone formation and recurrence.

In this study, we selected the normal human kidney proximal tubular epithelial cell line (HK-2 cell) as the research subject and stimulated it with CaOx monohydrate (COM) crystals to simulate the crystal–cell interaction. We applied transcriptome sequencing analysis (RNA-seq) to gain deeper insights into the molecular mechanisms behind this interaction. RNA-seq provides comprehensive insights into gene expression changes in HK-2 cells exposed to COM crystals, revealing the key pathways and molecular players involved in this interaction.

## 2. Materials and Methods

### 2.1. Cell Culture and Treatment

HK-2 cells were sourced from Procell (Wuhan, China) and cultured in DMEM/F12 medium (Gibco, Thermo Fisher Scientific, Waltham, MA, USA), supplemented with 10% fetal bovine serum (Gibco), at 37 °C in a humidified incubator with 5% CO_2_. The control group (n = 3) consisted of HK-2 cells without any treatment. In the experimental group (n = 3), HK-2 cells were exposed to 100 μg/mL of COM crystals (Macklin, C766516) for 24 h.

### 2.2. RNA Extraction, Library Construction, and Sequencing

Total cellular RNA was extracted from each sample using TRIzol reagent (Invitrogen, Carlsbad, CA, USA) following the manufacturer’s instructions [5]. RNA purity and quantity were assessed using a spectrophotometer (NanoDrop 1000, Thermo Fisher Scientific, Waltham, MA, USA) and confirmed by agarose gel electrophoresis. Eukaryotic mRNA was enriched using Oligo(dT) beads, and fragmentation buffer was added to break the mRNA into short fragments. The fragmented mRNA served as a template for synthesizing the first strand of cDNA using random hexamer primers. Subsequently, buffer, dNTPs, RNase H, and DNA polymerase I were used to synthesize the second strand of cDNA. The resulting double-stranded cDNA underwent end repair, poly(A) tail addition, and adaptor ligation. Magnetic beads were then used for purification and fragment selection. Finally, PCR amplification was performed to construct the library, which, after passing quality control, was sequenced on the Illumina Novaseq 6000 platform. RNA-seq services were provided by Bioyi Biotechnology Co., Ltd., Wuhan, China.

### 2.3. Bioinformatics Analysis

The sequencing data, referred to as raw reads, first underwent quality control to ensure their suitability for further analysis. Post-quality control, the raw reads were filtered to produce clean reads, which were then aligned to the reference genome. After alignment, the distribution and coverage of the reads on the reference genome were analyzed for a second round of quality control. Upon passing this step, gene expression profiles for each sample were generated using the Fragments Per Kilobase of transcript per Million mapped reads (FPKM) value.

### 2.4. Identification of Differentially Expressed Genes (DEGs)

Principal component analysis (PCA) was conducted to visualize differences among the samples. DEGs between the three control and three experimental samples were identified using the “DESeq2” package, with a threshold of *p*-value < 0.05 and |Log2 fold change (FC)| > 1 [6]. A volcano plot of the DEGs was generated using the “ggplot2” package, and a heatmap was created using the “pheatmap” package.

### 2.5. Functional Enrichment Analysis

Gene Ontology (GO) enrichment analysis was performed to investigate the potential functions of the DEGs [7]. GO terms were assessed across three categories: biological processes (BPs), molecular functions (MFs), and cellular components (CCs). Additionally, Kyoto Encyclopedia of Genes and Genomes (KEGG) enrichment analysis was conducted to identify DEG-related signaling pathways [8]. Gene set enrichment analysis (GSEA) was also carried out to explore functional differences between the two groups, using the gene set “c5.go.v2024.1.Hs.symbols.gmt” obtained from the MsigDB database (http://www.gsea-msigdb.org/gsea/msigdb/) accessed on 12 July 2024. Significant results were defined by an adjusted *p*-value < 0.05.

### 2.6. Construction of Protein–Protein Interaction (PPI) Network

The STRING database (http://string-db.org/) accessed on 12 July 2024 was utilized to construct the PPI network of the DEGs. To improve the reliability of PPI network, the interaction score was set to a high confidence level (>0.700). The number of adjacent nodes for each gene in the PPI network was calculated, and the top 30 genes with the highest number of adjacent nodes were visualized. Besides, the DEGs in the GO term “response to ERS” were regarded as ERS-related DEGs and subjected to PPI network construction.

### 2.7. Validation of DEGs in the Public Dataset

The public dataset GSE73680 (https://www.ncbi.nlm.nih.gov/geo/query/acc.cgi?acc=GSE73680) accessed on 12 July 2024 was used to validate the ERS-related DEGs. This dataset includes 24 renal papillary tissues from patients with CaOx stones and 6 normal renal papillary tissues from non-stone patients [9]. The Wilcoxon test was applied to verify the DEGs between the two groups.

### 2.8. Cell Viability and Apoptosis Assays

Cell viability was determined using Cell Counting Kit-8 (CCK-8, ABclonal, RM02823, Wuhan, China). First, 2 × 10^3^ cells were cultured in 96-well plates. After treatment with or without 100 μg/mL COM crystals for 24 h, 90 μL DMEM media with 10 μL CCK-8 was added to replace previous media in each well and incubated for another 4 h. The optical density values were detected at 450 nm using a microplate reader (Thermo Fisher Scientific, Waltham, MA, USA).

The apoptosis levels in HK-2 cells were assessed using an Annexin V-FITC Apoptosis Detection Kit (Beyotime, C1062S, Nanjing, China) through flow cytometry (CytoFLEX, Beckman Coulter, Brea, CA, USA). Following a 24 h treatment with or without 100 μg/mL COM crystals, the cells were washed three times with PBS, then incubated and stained with Annexin V-FITC and propidium iodide (PI) for 15 min at room temperature in the dark. The stained cells were subsequently analyzed via flow cytometry.

### 2.9. Quantitative Real-Time PCR (qRT-PCR)

We used TRIzol reagent (Invitrogen, Carlsbad, CA, USA) to extract total RNA and used a cDNA synthesis kit (Yeasen, 11141ES, Shanghai, China) to reverse transcribe the extracted RNA into cDNA. After that, we used SYBR Green Master Mix reagent (Yeasen, 11202ES, Shanghai, China) to perform qRT-PCR on a QuantStudio 6 Flex system. The relative RNA expression of genes was calculated using the 2^−ΔΔCt^ method by normalizing to β-actin expression. The sequences of primers used in the study were as follows: β-actin, forward: GTCATTCCAAATATGAGATGCGT, reverse: TGTGGACTTGGGAGAGGACT; IL6, forward: GTGTTGCCTGCTGCCTTCC, reverse: TCTGAAGAGGTGAGTGGCTGTC; HSPA5, forward: GCCGAGGAGGAGGACAAGAAG, reverse: AACACGCCGACGCAGGAG; ASNS, forward: AACAGTTCGTGCTTCAGTAGGTATG, reverse: GCGTAAGTTCATCTGATCCTTCTCC; CXCL8, forward: CTCTTGGCAGCCTTCCTGATTTC, reverse: GGGTGGAAAGGTTTGGAGTATGTC; ATF3, forward: GGCGACGAGAAAGAAATAAGATTGC, reverse: AGCCTTCAGTTCAGCATTCACAC; DDIT3, forward: GCTTGGCTGACTGAGGAGGAG, reverse: CTGACTGGAATCTGGAGAGTGAGG; XBP1, forward: GGATTCTGGCGGTATTGACTCTTC, reverse: CAGGCTGGCAGGCTCTGG; CEBPB, forward: CCACGGCCACGGACACC, reverse: GAGAAGAGGTCGGAGAGGAAGTC; EGR1, forward: TGGAGGAGATGATGCTGCTGAG, reverse: GCTGCTGCTGCTGCTGTTG; DNAJC3, forward: GCTGCTAAAGAAGTCCTCTCTGATC, reverse: TTGCCGCCGCCTCCTTG; CHAC1, forward: GAAACGACGGCGACCCTCAAG, reverse: CCAGAAACGGCGGCTGTAGC; FGF21, forward: GGTTTCTGTGCTGGCTGGTCTTC, reverse: CACCGTCCCATCCTCCCTGATC.

### 2.10. Western Blot (WB)

We used RIPA buffer (Boster, AR0102, Wuhan, China) containing 1% protease inhibitor PMSF (Boster, AR1192, China) to extract proteins from HK-2 cells. A BCA assay kit (Boster, AR1189, China) was used to measure the protein concentration. Equal amounts of protein were electrophoresed in 10% SDS-PAGE gels and transferred to PVDF membranes (Millipore, Burlington, MA, USA). After being blocked with 5% bovine serum albumin at room temperature for 2 h, the membranes were incubated with the primary antibodies against OPN (Proteintech, 22952-1-AP, Wuhan, China), CHAC1 (ABclonal, A15584, Wuhan, China), FGF21 (ABclonal, A3908, Wuhan, China), and β-actin (Proteintech, 66009-1-Ig, Wuhan, China) at 4 °C overnight. The membranes were then incubated with the appropriate horseradish peroxidase-conjugated secondary antibodies at room temperature for 2 h. Finally, the protein bands were visualized with an ECL kit (Yeasen, 36208ES, Shanghai, China). The relative protein expression of genes was quantified using ImageJ software (version 1.8.0.) by normalizing to β-actin expression.

## 3. Results

### 3.1. Validation of the Cell–Crystal Interaction Model

**Figure 1:**
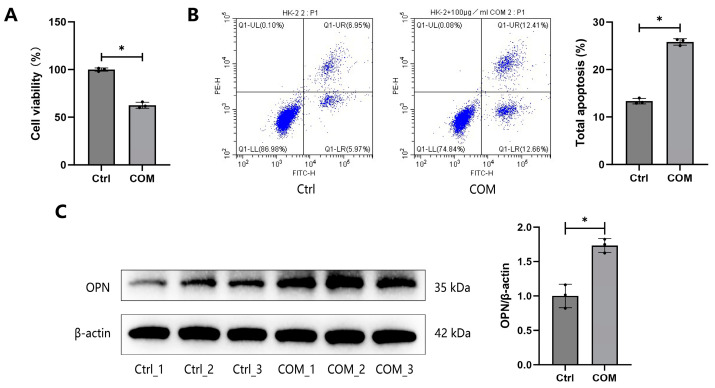
**Validation of the cell–crystal interaction model.** (**A**) The cell viability of HK-2 cells was assessed by CCK-8 assay after treatment with 100 μg/mL COM for 24 h. (**B**) The apoptosis levels of HK-2 cells were detected by flow cytometry after treatment with 100 μg/mL COM for 24 h. (**C**) The expressions of OPN proteins were detected by WB in HK-2 cells after treatment with 100 μg/mL COM for 24 h. * represents *p* < 0.05.

We first investigated the effects of COM crystals on cell viability and apoptosis. The CCK-8 assay demonstrated a significant reduction in the viability of HK-2 cells exposed to COM crystals (Figure 1A). Additionally, flow cytometry revealed a marked increase in the apoptosis levels of COM-treated cells compared to untreated controls (Figure 1B). Moreover, the expression of the stone-related marker OPN was up-regulated in COM-treated cells (Figure 1C). These findings confirmed the successful establishment of the cell–crystal interaction model.

### 3.2. Alignment Statistic of Reads Aligned with Reference Genome

Table 1 shows the sequencing results of an HK-2 cellular RNA library; approximately 42.2–50.1 M clean reads of each library were obtained. The percentage of uniquely mapped reads ranged from 87.85 to 88.51%, while the percentage of multiple mapped reads ranged from 8.71 to 9.33%.

### 3.3. Identification of DEGs

**Figure 2:**
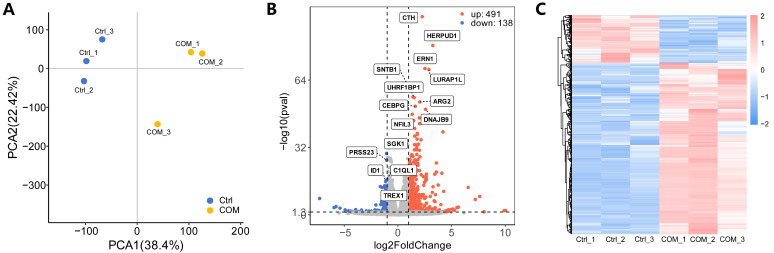
**Identification of DEGs.** (**A**) The PCA plot showing the distribution of the two groups. (**B**) The volcano plot of DEGs. (**C**) The heatmap of DEGs.

The PCA plot shows that the control group is clearly separated from the COM group, suggesting a clear separation between the two groups (Figure 2A). A total of 629 DEGs were identified, of which 491 were up-regulated and 138 were down-regulated. The list of the 629 DEGs were presented in Table S1. The volcano plot and the heatmap were used to visualize DEGs (Figure 2B,C). The heatmap with the detailed gene list is given in Figure S1.

### 3.4. Functional Enrichment Analysis

**Figure 3:**
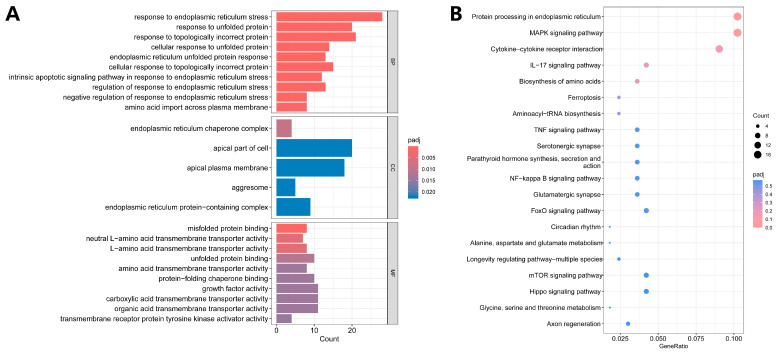
GO and KEGG functional enrichment analysis. (**A**) GO analysis of DEGs. (**B**) KEGG analysis of DEGs.

DEGs were subjected to GO and KEGG enrichment analysis to explore the potential function and pathways during the crystal–cell interaction. The results of GO enrichment analysis are presented as three categories in Figure 3A: biological process (BP), cellular component (CC), and molecular function (MF). The top five enriched biological processes were as follows: response to endoplasmic reticulum stress (ERS), response to unfolded proteins, response to topologically incorrect proteins, cellular response to unfolded proteins, and endoplasmic reticulum (ER) unfolded protein response (UPR). The top five enriched cellular components were the ER chaperone complex, the apical part of the cell, the apical plasma membrane, the aggresome, and the ER protein-containing complex. The top five enriched molecular functions were misfolded protein binding, neutral L-amino acid transmembrane transporter activity, L-amino acid transmembrane transporter activity, unfolded protein binding, and amino acid transmembrane transporter activity. The detailed results of GO enrichment analysis are presented in Table S2. The results of KEGG enrichment analysis showed that DEGs were related to protein processing in the endoplasmic reticulum, MAPK signaling pathway, cytokine–cytokine receptor interaction, IL-17 signaling pathway, biosynthesis of amino acids, and ferroptosis (Figure 3B). 

**Figure 4:**
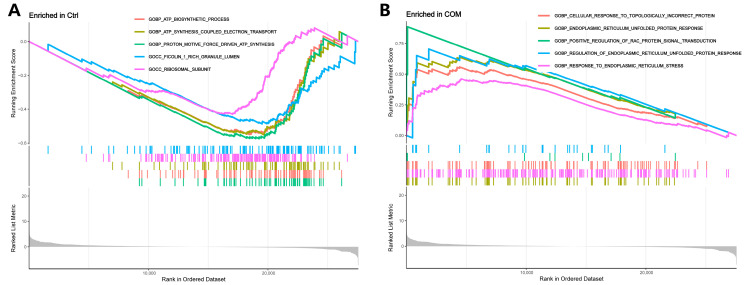
**GSEA functional enrichment analysis.** (**A**) GSEA showing the enriched GO terms in the control group. (**B**) GSEA showing the enriched GO terms in the experimental group.

We also applied GSEA to explore the functional differences between the two groups. Proton motive force-driven ATP synthesis, ATP biosynthetic processes, and ATP synthesis coupled electron transport were enriched in the control group (Figure 4A). The endoplasmic reticulum UPR, response to ERS, and cellular response to topologically incorrect proteins were enriched in the COM group (Figure 4B). It can be seen that ERS seems to play an important role in the interaction between CaOx crystals and RTECs.

### 3.5. Construction of PPI Network

**Figure 5:**
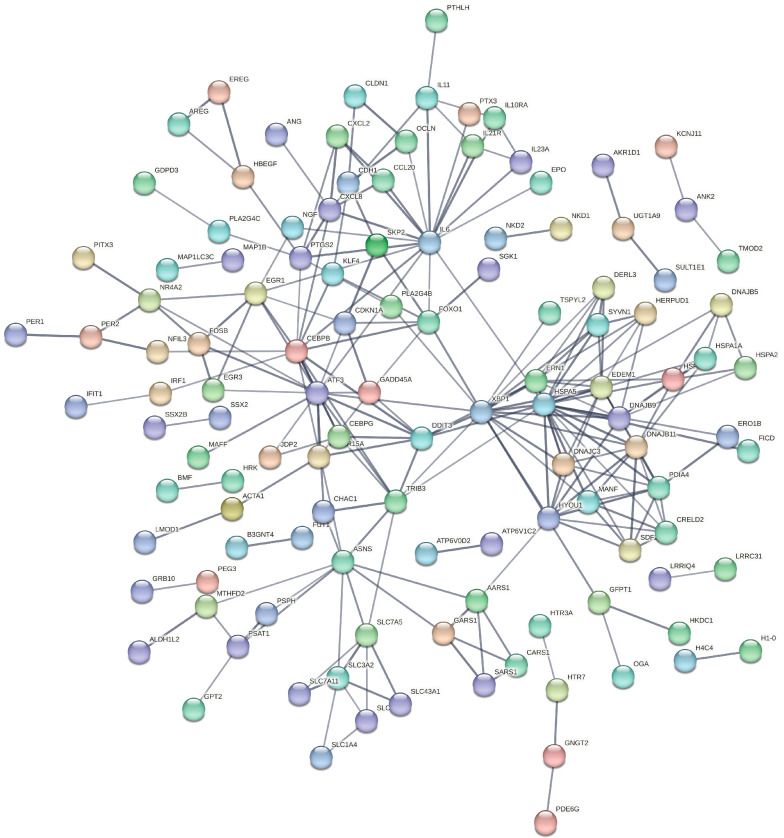
PPI network of all DEGs.

**Figure 6:**
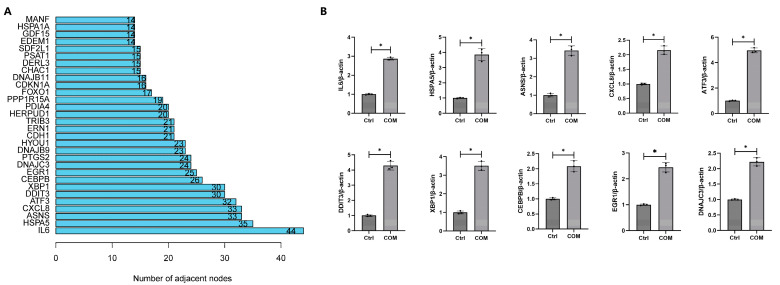
**Validation of transcriptome sequencing results.** (**A**) The top 30 DEGs with the largest number of adjacent nodes. (**B**) The expression of the top 10 DEG mRNAs were detected by qRT-PCR in HK-2 cells after treatment with 100 μg/mL COM for 24 h. * represents *p* < 0.05.

All DEGs were imported to the STRING database to construct the PPI network (Figure 5). The top 30 DEGs with the largest number of adjacent nodes were presented in Figure 6A. The top 10 DEGs with the largest number of adjacent nodes (IL6, HSPA5, ASNS, CXCL8, ATF3, DDIT3, XBP1, CEBPB, EGR1, and DNAJC3) were selected for qRT-PCR validation. RNA-seq analysis revealed that all of these genes were up-regulated in COM-treated cells. The qRT-PCR results confirmed that the expression trends of the top 10 DEGs were consistent with the RNA-seq data, indicating the reliability of the transcriptome sequencing results (Figure 6B).

### 3.6. Validation of DEGs

**Figure 7:**
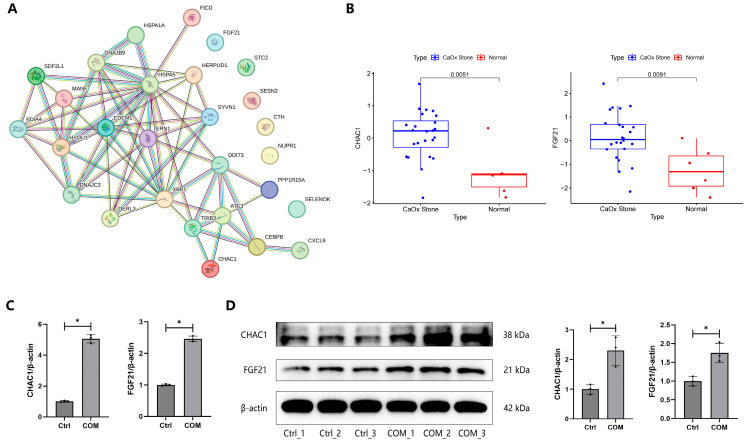
**Validation of DEGs.** (**A**) PPI network of ERS-related DEGs. (**B**) The expression of CHAC1 and FGF21 mRNAs between CaOx renal papillary tissues and normal renal papillary tissue. (**C**) The expressions of the CHAC1 and FGF21 mRNAs were detected by qRT-PCR in HK-2 cells after treatment with 100 μg/mL COM for 24 h. (**D**) The expressions of the CHAC1 and FGF21 proteins were detected by WB in HK-2 cells after treatment with 100 μg/mL COM for 24 h. * represents *p* < 0.05.

We found that 20 of the top 30 DEGs were related to the GO term “response to ERS”, including HSPA5, CXCL8, ATF3, DDIT3, XBP1, CEBPB, DNAJC3, DNAJB8, HYOU1. ERN1, TRIB3, HERPUD1, PDIA4, PPP1R15A, CHAC1, DERL3, SDF2L1, EDEM1, HSPA1A, and MANF. We then extracted the 28 ERS-related DEGs from the GO term “response to ERS” for PPI network construction. Apparently, there was a close interaction among these genes (Figure 7A). Then, we used the public dataset GSE73680 to validate ERS-related DEGs. Table 2 shows the expression levels of 28 ERS-related genes in COM-treated cells from our RNA-seq and CaOx renal papillary tissues from the public dataset. We found that glutathione-specific gamma-glutamylcyclotransferase 1 (CHAC1) and fibroblast growth factor 21 (FGF21) were up-regulated in both COM-treated cells and CaOx renal papillary tissues (Figure 7B). qRT-PCR and WB results confirmed that CHAC1 and FGF21 were significantly up-regulated in COM-treated cells compared to untreated controls (Figure 7C,D), suggesting that they might be critical players in ERS-mediated CaOx stone formation.

## 4. Discussion

The interaction between CaOx crystals and RTECs is a complex biological process. This study aimed to explore the molecular changes during this interaction using transcriptomics. We first observed significant differences between HK-2 cells stimulated by CaOx crystals and untreated cells, identifying 629 DEGs. Functional enrichment analysis suggested that ERS might play a critical role in this interaction. Additionally, we confirmed that ERS-related DEGs, including CHAC1 and FGF21, were significantly up-regulated in CaOx renal papillary tissues.

The ER is a critical organelle involved in protein folding, lipid synthesis, and calcium storage. ERS is typically triggered by the accumulation of misfolded or unfolded proteins within the ER lumen, disrupting ER homeostasis and function [10]. In response, cells activate the UPR, a protective signaling pathway designed to restore ER function by enhancing protein folding capacity, reducing new protein synthesis, and promoting the degradation of misfolded proteins [11]. However, if ERS is prolonged or unresolved, it can lead to cellular damage and death. The UPR operates through three primary signaling pathways mediated by the sensors inositol-requiring enzyme 1 (IRE1), protein kinase RNA-like ER kinase (PERK), and activating transcription factor 6 (ATF6) [12].

IRE1 (also known as ERN1) is an ER transmembrane sensor with both kinase and endoribonuclease activity [13]. Upon activation, IRE1 splices X-box binding protein 1 (XBP1) mRNA, creating a potent transcription factor that up-regulates genes involved in protein folding, secretion, and ER-associated degradation [14]. In our study, we observed the increased expression of IRE1 and XBP1 in HK-2 cells exposed to CaOx crystals, suggesting that IRE1 activation likely plays a role in enhancing the protein folding capacity of RTECs as they respond to the CaOx crystal-induced ERS. PERK can phosphorylate eukaryotic initiation factor 2α (eIF2α), leading to a global reduction in protein synthesis and thereby decreasing the influx of nascent proteins into the ER. Concurrently, PERK activation promotes the expression of activating transcription factor 4 (ATF4), which regulates genes involved in amino acid metabolism and redox balance, facilitating cellular adaptation to stress. When ER stress is severe, ATF4 also induces the expression of DNA damage inducible transcript 3 (DDIT3), a pro-apoptotic transcription factor that contributes to cell death [15]. Our results showed that PERK and ATF4 were moderately up-regulated but did not reach the threshold for screening DEGs. However, DDIT3, the key downstream targets of the PERK- eIF2α-ATF4 pathway, was found to be significantly up-regulated in the CaOx crystal-stimulated HK-2 cells. This activation of DDIT3 might contribute to the initiation of cell apoptosis, which may facilitate CaOx crystal adhesion and exacerbate kidney stone formation. ATF6 can translocate to the Golgi apparatus, where it undergoes cleavage to produce an active transcription factor that enhances the synthesis of chaperones and components of the ER-associated degradation system [16]. We also observed a slight increase in the expression of ATF6, which was moderately up-regulated but did not reach the threshold. Together, these pathways work synergistically to alleviate ERS and restore protein homeostasis within the ER. However, if stress persists, the UPR may shift from a protective to a maladaptive role, potentially contributing to the pathogenesis of various diseases. Our study revealed that the activation of the IRE1 and PERK pathways may play an important role in CaOx crystal-induced ERS.

One notable observation from the PPI network analysis was that IL6 exhibited the largest number of adjacent nodes. IL6 is a key pro-inflammatory cytokine [17], and its prominence in the network indicates its central role in mediating the inflammatory response triggered by CaOx crystal exposure. ERS can stimulate increased production and secretion of IL6, and, in turn, IL6 can amplify ERS by promoting inflammation [18]. The up-regulation of IL6 in our study supports that inflammation is a critical aspect of the cellular response to CaOx crystals, potentially exacerbating ERS and further contributing to cellular damage. After IL6, HSPA5 emerged as the DEG with the second-highest number of adjacent nodes. Under non-stressed conditions, HSPA5 binds to the UPR sensors IRE1, PERK, and ATF6, maintaining them in an inactive state. Under ERS, HSPA5 releases its hold on the UPR sensors and preferentially binds to unfolded or misfolded proteins, initiating the activation of IRE1, PERK, and ATF6 [19]. Numerous studies have confirmed that HSPA5 was one of the most highly up-regulated proteins during ERS [20], which aligns with our findings.

Some studies have unraveled the mysterious veil of ERS in kidney stone formation. Abhishek et al. found that exposure to oxalate triggered oxidative stress to induce ERS and further activate apoptosis [21]. The use of 4-phenylbutyrate (4-PBA), a small chemical chaperone known as an ERS inhibitor, has been shown to protect against hyperoxaluria-induced renal injury and inflammation [22,23]. Sharma et al. found that hyperoxaluria-induced ERS initiated mitochondria dysfunction via alteration in the sigma-1 receptor protein located in the mitochondria-associated ER membranes, leading to renal injury and crystal deposition [24]. Hyperoxaluria-induced ERS also can mediate excessive autophagy via the PERK- eIF2α-ATF4 pathway to promote stone formation [25]. Sun et al. were the first to investigate the role of ERS in HK-2 cells exposed to CaOx crystals [26]. They found that the damage and apoptosis of HK-2 cells stimulated by CaOx crystals are closely related to the level of ERS and that the inhibition of ERS mitigated these adverse effects. However, to date, no studies have utilized transcriptomic approaches to explore the effects of ERS on the crystal–cell interaction.

Our study not only identified the involvement of ERS and UPR in the crystal–cell interaction but also observed increased expression of two ERS-related DEGs, CHAC1 and FGF21, in renal papillary tissues from CaOx stone patients. CHAC1 is up-regulated in response to ERS through the activation of the PERK-eIF2α-ATF4 pathway [27]. CHAC1 is also linked to the activation of the pro-apoptotic transcription factor DDIT3, further contributing to cell death [28]. CHAC1 is capable of degrading glutathione, a crucial antioxidant that helps maintain cellular redox balance. The reduction in glutathione levels due to CHAC1 activity exacerbates oxidative stress, pushing the cell towards apoptosis [29]. In addition, glutathione is required for the activity of glutathione peroxidase 4 (GPX4), an enzyme that protects cells from lipid peroxidation. Glutathione depletion diminishes GPX4 activity, leading to the accumulation of lipid peroxides and triggering ferroptosis [30]. KEGG analysis in our study also showed that DEGs were significantly enriched in ferroptosis. Thus, CHAC1 could be a potential therapeutic target in CaOx stone disease. Modulating CHAC1 activity may help mitigate the damaging effects of prolonged ERS by preserving cellular glutathione levels and preventing apoptosis and ferroptosis. FGF21 expression can be induced via ATF4- and DDIT3-dependent pathways [31]. The increase in FGF21 acts as an adaptive response to alleviate ERS and protect cells from apoptosis by enhancing mitochondrial function, reducing oxidative stress, and improving metabolic homeostasis [32,33]. Additionally, FGF21 influences cellular processes such as autophagy, promoting the clearance of misfolded proteins and damaged organelles, thereby alleviating the burden on the ER [34]. Thus, enhancing FGF21 signaling could offer a strategy to counteract the detrimental effects of ERS.

Several limitations of our study should be acknowledged. Firstly, the sequencing results require further validation at both the cellular and animal levels to confirm their relevance and accuracy. Secondly, our study is primarily descriptive; therefore, additional experimental investigations are needed to elucidate the biological functions and specific mechanisms of the DEGs in CaOx stone formation.

## 5. Conclusions

Our study provides novel insights into the role of ERS in the interaction between CaOx crystals and RTECs. We identified significant changes in the expression of ERS-related DEGs and validated the expression of CHAC1 and FGF21 in renal papillary tissues from CaOx stone patients. CHAC1, through its role in glutathione degradation and promotion of ferroptosis, and FGF21, by modulating mitochondrial function and autophagy, emerge as critical players in the pathogenesis of CaOx stones.

## Figures and Tables

**Table 1:** Alignment statistic of reads aligned with reference genome.

Sample Name	Total Reads	Total Mapped (%)	Uniquely Mapped (%)	Multiple Mapped (%)
Ctrl_1	47,390,414	45,949,461 (96.96%)	41,796,275 (88.20%)	4,153,186 (8.76%)
Ctrl_2	42,219,656	41,042,552 (97.21%)	37,366,844 (88.51%)	3,675,708 (8.71%)
Ctrl_3	49,909,816	48,541,929 (97.26%)	43,884,870 (87.93%)	4,657,059 (9.33%)
COM_1	50,143,150	48,635,137 (96.99%)	44,239,250 (88.23%)	4,395,887 (8.77%)
COM_2	43,001,784	41,721,455 (97.02%)	37,877,062 (88.08%)	3,844,393 (8.94%)
COM_3	47,134,688	45,793,724 (97.16%)	41,409,278 (87.85%)	4,384,446 (9.30%)

**Table 2:** The expression levels of 28 ERS-related genes in COM-treated cells from our RNA-seq and CaOx renal papillary tissues from the public dataset.

ERS-Related DEGs	Our RNA-Seq Data		Public Dataset GSE73680	
	Log_2_ FC (the COM Groups Versus the Control Group)	*p*-Value	Log_2_ FC (CaOx Renal Papillary Tissues Versus Normal Renal Papillary Tissues)	*p*-Value
ATF3	3.7524	0.000000	−0.0216	0.972784
CEBPB	1.0315	0.000000	−0.6940	0.165201
CHAC1	2.6641	0.000003	1.1802	0.001305
CTH	2.2632	0.000000	0.0204	0.959321
CXCL8	1.7993	0.000000	0.4042	0.655936
DDIT3	3.5863	0.000000	−0.7056	0.048815
DERL3	1.9735	0.000000	0.6974	0.038880
DNAJB9	2.572	0.000000	−0.6196	0.048469
DNAJC3	1.7623	0.000000	0.1448	0.559762
EDEM1	1.0806	0.000000	−0.2502	0.539455
ERN1	2.5113	0.000000	0.0381	0.884129
FGF21	5.632	0.000253	1.4001	0.003260
FICD	2.0538	0.000000	0.2295	0.441651
HERPUD1	3.2536	0.000000	−0.8134	0.020813
HSPA1A	−1.0059	0.000000	−0.8592	0.020964
HSPA5	2.479	0.000000	−0.7918	0.040477
HYOU1	1.7581	0.000000	−0.0876	0.719632
MANF	1.8644	0.000000	−0.1799	0.520851
NUPR1	1.9967	0.000000	−0.7957	0.069520
PDIA4	1.008	0.000000	−0.2344	0.521433
PPP1R15A	2.2295	0.000000	−0.4684	0.405668
SDF2L1	2.5809	0.000000	0.2598	0.420405
SELENOK	1.0897	0.000000	−0.2884	0.263865
SESN2	2.0397	0.000000	0.1554	0.538660
STC2	1.3444	0.000000	0.3658	0.391225
SYVN1	1.3592	0.000000	−0.7824	0.064149
TRIB3	2.6531	0.000000	0.6137	0.101077
XBP1	1.5692	0.000000	−0.1643	0.697346

## Data Availability

Data are contained within the article.

## Supplementary Materials

The following supporting information can be downloaded at: https://www.mdpi.com/article/10.3390/biology13100774/s1, Figure S1: The heatmap with the detailed gene list; Table S1: The list of the 629 DEGs; Table S2: The detailed results of GO enrichment analysis.
