# Supplementary material for: The Involvement of Endoplasmic Reticulum Stress during the Interaction between Calcium Oxalate Crystals and Renal Tubular Epithelial Cells
Source: Biology (Basel). 2024 Sep 27;13(10):774. doi: 10.3390/biology13100774 (PMC11504059; doi:10.3390/biology13100774)
Supplement: Supplementary file 1 [file biology-13-00774-s001.zip › Table S1.pdf]

Table S1. The list of the 629 DEGs.

Gene ID	Ctrl read count	COM read count	Log2 Fold Change	P-value
CTH	562.4114001	2700.130106	2.2632	7.32E-95
HERPUD1	1813.415031	17298.43388	3.2536	3.69E-81
ERN1	199.9271702	1139.506381	2.5113	3.29E-70
LURAP1L	143.4064376	1061.036603	2.8848	9.05E-70
UHRF1BP1	1822.716744	4822.096831	1.4035	1.18E-56
SNTB1	1735.201273	4969.020175	1.5173	2.46E-56
ARG2	476.4781849	1956.474408	2.0383	2.77E-54
CEBPG	689.6766204	2125.968155	1.6232	2.86E-52
DNAJB9	329.5650238	1960.046696	2.572	7.67E-51
NFIL3	814.6242101	2055.777134	1.3347	1.63E-48
NUPR1	222.0189129	885.9097457	1.9967	8.79E-47
ITPKA	340.8981251	2093.981785	2.6185	1.06E-46
PSPH	1460.683614	4075.776156	1.4804	1.41E-45
SESN2-2	958.5965582	3905.607806	2.026	3.96E-44
SESN2	931.6647045	3832.305993	2.0397	4.67E-44
XBP1	3310.436326	9825.091034	1.5692	1.06E-41
INHBE	286.2375815	5239.935249	4.1934	4.63E-40
HKDC1	8458.667186	29704.03654	1.8121	2.30E-39
CXCL8	597.8918424	2081.863258	1.7993	8.72E-39
H1-0	639.5015347	1890.019533	1.5632	4.29E-38
ALDH1L2	343.3211715	1185.337258	1.7867	1.08E-36
SLC3A2	4635.267704	20664.69925	2.1563	2.57E-36
TNFRSF9	474.6211695	1786.091415	1.9115	2.78E-34
SLC22A15	452.8479569	1338.870335	1.5625	4.76E-34
ERO1B	289.2850722	997.0994572	1.7848	6.11E-34
PCK2-2	562.725033	1937.860293	1.7834	6.02E-34
PCK2	618.5988959	2100.216016	1.7629	6.27E-33
MTHFD2	2333.979356	7603.662388	1.7036	1.20E-31
HOXB9	715.468466	2084.7378	1.5424	1.37E-31
TUBE1	207.4704722	825.6870321	1.9914	2.01E-31
SGK1	5057.348585	2425.123294	-1.0602	7.27E-30
DNAJC3	1006.195334	3413.110212	1.7623	2.03E-29
SGPP2	477.8188063	1176.721831	1.2991	2.36E-29
LMO4	766.8224818	1800.616976	1.231	1.14E-28
LARP6	778.0810136	2051.129965	1.3974	1.59E-28
FICD	302.9739484	1259.298981	2.0538	2.58E-28
NHSL1	390.7225663	1432.562136	1.8737	5.58E-27
SLC35F6	3118.396453	6836.405335	1.1322	5.92E-27
RNF41	1362.741663	4061.977336	1.5754	7.27E-27
SLC1A4	1024.937195	3095.837428	1.5944	9.06E-27
PRSS23	8808.161767	4284.969226	-1.0395	1.26E-26

SLC39A14	3165.479968	10192.78194	1.6869	1.31E-26
CBX4	755.965538	2094.877788	1.4699	8.89E-26
PSAT1	1485.824102	3193.402321	1.1035	1.18E-25
AKNA	1822.027718	5738.752417	1.655	2.00E-25
DDIT3	1400.117408	16818.16373	3.5863	2.38E-25
RCAN1	372.8888229	1345.802709	1.8513	3.29E-25
DUSP16	464.4496003	1047.133916	1.1727	3.53E-25
GPT2	2581.447242	5814.440684	1.1711	5.48E-25
PIGA	583.9634873	1251.048596	1.0987	6.13E-25
GTPBP2	4038.074532	8588.983369	1.0887	1.29E-24
DUSP16-2	397.4100664	884.027754	1.1533	2.22E-24
MANF	1129.765351	4114.378378	1.8644	3.17E-24
GARS1	5561.943661	11831.03535	1.0888	3.76E-24
JDP2	545.2952116	1154.667774	1.0827	6.65E-24
SNHG1	1261.464125	2764.493654	1.1315	6.87E-24
CRELD1	470.3260934	1301.816403	1.4679	1.04E-23
GFPT1	3741.25651	8015.023942	1.0992	2.41E-23
CARS1	935.858064	2240.698741	1.2592	6.25E-23
AARS1	6359.662896	14475.65862	1.1865	9.43E-23
ARHGEF2	2302.984732	6134.854546	1.4134	2.43E-22
CARS1-2	770.9343436	1850.127901	1.2625	2.60E-22
HYOU1	3077.0736	10409.15434	1.7581	4.23E-22
SLC7A1	3689.956743	10037.26839	1.4435	5.00E-22
PTX3	116.4414579	471.6341	2.0144	9.35E-22
TMEM50B	234.8098934	696.8024725	1.5683	1.25E-21
HYOU1-2	2769.422725	9271.052392	1.743	1.81E-21
SLC6A9	159.6736502	1926.841297	3.5917	4.19E-21
EDEM1	1876.573218	3969.043934	1.0806	6.87E-21
ZNF804A	7.384663038	126.9398179	4.0926	1.60E-20
MSTO1	1060.429415	3116.449813	1.5549	2.05E-20
SH3TC1	1279.519709	3143.980909	1.2966	3.64E-20
BEX2	56.43660088	357.5369234	2.6574	4.15E-20
SLC7A11	200.0991455	2164.400279	3.4343	4.89E-20
XPOT	5497.360376	11075.01115	1.0104	5.00E-20
GRB10	681.634683	1942.898346	1.5103	5.54E-20
IL21R	80.56199882	361.6611313	2.1643	8.02E-20
LRRC49	74.71677779	238.280246	1.6738	2.66E-19
SYVN1	1635.323968	4195.988916	1.3592	3.35E-19
APTR	267.7150493	686.7039322	1.358	3.50E-19
ULBP1	21.24993615	132.356718	2.6342	5.81E-19
HRK	436.7743089	1025.163468	1.2294	6.52E-19
SLC43A1	41.25450445	213.922862	2.3668	7.53E-19
TMEM50B-2	180.1578986	506.1868292	1.4896	8.35E-19
PER1	970.8058995	2772.067904	1.5135	1.07E-18

NFXL1	604.6445841	1310.198271	1.1153	1.14E-18
IRF1	1363.455445	3517.936363	1.3672	1.68E-18
TES	1761.633289	3873.024777	1.1366	1.80E-18
ATP6V0E2-AS1	173.633099	434.7480025	1.3213	2.23E-18
TRIM2	871.4671812	1752.945355	1.008	8.39E-18
TRIB3	3807.881683	23953.59059	2.6531	9.21E-18
CEBPB	3046.921745	6229.775001	1.0315	1.02E-17
CXCL2	229.850549	756.3775962	1.7169	1.52E-17
SDF2L1	1109.686391	6640.067967	2.5809	1.83E-17
SLC33A1	1456.610796	2932.774827	1.0094	2.43E-17
C1QL1	1263.719025	622.5810673	-1.0207	2.64E-17
CDKN1A	2372.764651	5002.835848	1.0758	3.14E-17
SARS1	4683.663118	10257.16492	1.1308	4.23E-17
MAP1B	588.3377923	1540.625427	1.3878	4.89E-17
TM6SF1	33.24019242	201.2852377	2.5982	5.15E-17
MIA2	907.0493925	1918.785966	1.0813	7.20E-17
DNAJB11	3431.141391	7867.496724	1.1971	8.39E-17
GZF1	879.6182463	1803.380932	1.0351	9.40E-17
ID1	2460.215578	1107.501703	-1.1509	1.69E-16
HSPA5	22978.69795	128109.2698	2.479	3.60E-16
EXOSC6	1039.234075	2641.882526	1.3455	3.83E-16
IFRD1	1075.687817	2488.143189	1.2092	3.82E-16
CAMTA2	669.4857884	1909.800623	1.5116	1.23E-15
SNHG17	468.2448449	1402.654375	1.5821	1.78E-15
DNAJB5	328.8596726	658.1139306	1.0007	3.40E-15
ZFAND2A	360.0249509	983.1303903	1.4486	3.82E-15
ATF3	566.0695973	7630.253289	3.7524	6.83E-15
RAPH1	564.0516014	1356.719171	1.2664	8.26E-15
ASNS	565.6829584	3006.759188	2.4099	9.78E-15
SELENOK	451.8691001	961.9481811	1.0897	1.25E-14
TMEM268	755.4042933	1701.219721	1.1706	1.40E-14
TREX1	326.5998091	138.282876	-1.2373	1.47E-14
MAFF	1107.698483	3043.953123	1.4579	2.18E-14
NUAK2	2085.885934	4553.001677	1.1261	2.84E-14
DHRS9	662.8153191	212.3204568	-1.6411	3.93E-14
LINC01881	53.3867155	192.536627	1.8503	4.07E-14
KCNJ11	8.025913339	78.3982268	3.2747	4.74E-14
HSPA1A-5	1889.731101	906.7178533	-1.0592	4.99E-14
IFIT1	457.152482	216.6296339	-1.0775	5.73E-14
KLHDC7B	4.264278322	403.1926916	6.5458	9.24E-14
STC2	2701.797079	6861.686876	1.3444	9.27E-14
ZNF488	249.1361325	91.24277267	-1.4472	1.00E-13
LOC100294145-8	111.619941	279.7601397	1.324	1.27E-13
ZNF674	124.4929021	333.2541909	1.4178	1.46E-13

XRRA1	161.727095	365.7775165	1.1777	1.91E-13
LOC100294145	107.5348708	273.2937386	1.3442	2.22E-13
LOC100294145-2	110.9208682	275.0950448	1.3088	3.09E-13
SKP2	1277.707592	574.0666927	-1.1532	3.63E-13
ZNF165	112.3730707	338.3488124	1.5901	4.76E-13
LINC02970	84.15178025	299.711545	1.829	5.81E-13
CLDN1	18137.71038	41922.77183	1.2087	8.94E-13
LOC100294145-4	106.2941675	256.1925305	1.2681	9.72E-13
LOC100294145-3	109.2438947	266.2270668	1.2834	1.07E-12
LOC100294145-7	107.2044988	261.3249035	1.2837	1.15E-12
HNF1B-2	1626.95136	793.0805783	-1.0364	1.26E-12
C17orf100	53.28655797	192.1850397	1.8468	1.43E-12
LOC100294145-5	111.0044627	266.2936091	1.2609	1.52E-12
HSPA1A-3	1231.747757	598.1805417	-1.0417	1.74E-12
TUFT1	1519.851624	3240.575974	1.0924	4.05E-12
ZFP69B	82.23711779	273.563918	1.7301	6.89E-12
LAMP3	3.690597323	71.52465707	4.2653	7.16E-12
SNHG11	205.759056	433.3248799	1.0722	7.60E-12
SYTL1	137.7377353	357.5071787	1.3744	7.66E-12
SNHG15	779.222403	1714.589606	1.1371	1.06E-11
FOXO6-2	320.8210812	655.5674125	1.0317	1.51E-11
HSPA1B-3	1002.432922	497.0171315	-1.0116	1.61E-11
LOC100294145-6	127.0070176	303.2647909	1.2526	1.73E-11
HSPA1B-5	660.0554493	324.0763841	-1.0258	1.81E-11
HSPA1B	558.208116	269.7574368	-1.049	1.99E-11
ANK2	393.830153	986.6748832	1.3245	2.05E-11
NGF	12.33826734	114.7057677	3.2058	2.19E-11
LARP1B	450.7661682	1001.852614	1.1523	2.36E-11
BMF	809.5528045	335.3701812	-1.2706	2.75E-11
DUSP8	53.87333358	173.5621939	1.6855	3.49E-11
IER3-2	421.2681196	1144.993183	1.4414	3.86E-11
HNF1B	2460.166682	1217.319066	-1.0149	5.31E-11
HSPA1A	654.860567	325.8914076	-1.0059	9.10E-11
MST1	126.849037	263.708535	1.055	1.01E-10
HBEGF	325.9446858	693.5084112	1.0883	1.10E-10
USP2	463.4310985	213.9355096	-1.1163	1.20E-10
CCDC163	148.10872	335.0363113	1.1769	1.22E-10
RAB7B	229.9811425	100.760262	-1.187	1.49E-10
LOC101927506	14.64794044	75.01358889	2.3589	2.22E-10
IER3-5	434.1055618	1128.14647	1.3768	2.30E-10
LOC124905564	133.4205961	278.2699229	1.0581	2.45E-10
PPP1R15A	1561.090825	7321.466005	2.2295	2.46E-10
LCN12	37.89948138	123.1661613	1.7059	2.58E-10
DERL3	29.83057464	117.1731509	1.9735	2.79E-10

KLF15	19.99721375	97.89720937	2.3017	2.86E-10
LOC124904731	5.858853397	65.75627198	3.5019	2.87E-10
SDCCAG8-2	251.2361609	503.283899	1.0019	3.01E-10
DUSP8-3	44.68977366	138.0427412	1.6248	3.45E-10
DUSP8-2	43.60250642	136.0556174	1.6385	3.72E-10
EML2	221.0695949	504.9376911	1.1902	5.67E-10
NR4A2	23.1959907	99.43055426	2.0941	5.97E-10
TSPYL2	1383.966297	2908.440656	1.0715	7.10E-10
IER3-6	374.1129644	979.3012344	1.3871	9.99E-10
IER3-3	371.0280986	972.1577953	1.3885	1.07E-09
IER3-4	366.6544537	962.2869265	1.3909	1.09E-09
GOLGA4-AS1	131.6103386	281.5697866	1.0964	1.14E-09
SLC7A5	2526.030357	12374.42132	2.2923	1.17E-09
IER3	272.0618472	730.7727844	1.4239	1.42E-09
PDIA4	9204.327627	18511.13334	1.008	1.86E-09
LOC124905606	6.422787586	51.94047439	3.0144	2.00E-09
MINDY4	0	37.03287566	7.6288	2.03E-09
ETV5	1944.858128	3950.399775	1.022	2.38E-09
SYS1-DBNDD2	46.78938431	194.0890859	2.0585	3.12E-09
GADD45A	1184.685233	4201.447986	1.8262	3.28E-09
SHE	124.6018509	254.6949271	1.0311	3.29E-09
AREG	1209.337371	7341.751728	2.6018	4.30E-09
DUSP8-4	40.93788546	125.8383199	1.6171	6.15E-09
GDF15	5481.623319	17067.95644	1.6386	7.26E-09
DERL3-2	20.69509041	88.86525378	2.1027	7.52E-09
TMEM184A	1021.051784	2122.603378	1.0552	7.57E-09
PER2	163.1211145	344.8429581	1.0775	1.12E-08
CPLANE2	81.31476735	192.6084538	1.2407	1.33E-08
FAM110D	35.89387043	142.6321149	1.9847	1.34E-08
LONRF2	123.2305541	326.0516064	1.4002	1.57E-08
CASC15	50.79594226	145.4811401	1.5191	1.73E-08
CPLANE2-2	63.86826348	163.754501	1.3555	1.77E-08
DDR2	10.26454874	57.24516569	2.4875	1.83E-08
INMT-MINDY4	28.07347503	0	-7.2902	1.89E-08
CCDC171	75.28232588	179.0399496	1.2469	2.09E-08
MOCS2-DT	37.8084123	108.9623811	1.5246	2.60E-08
NRXN3	76.87008477	171.3155517	1.1532	2.79E-08
S1PR5	234.1405988	106.8809805	-1.1282	4.71E-08
LOC124901152	0	27.75340869	7.2119	5.09E-08
PARD6A	91.14651376	212.9816752	1.2292	7.77E-08
LINC00662	59.79228173	170.2474984	1.5082	8.13E-08
GDF6	179.7588812	80.25680433	-1.1635	8.64E-08
EML2-AS1	45.71066774	138.6119009	1.6027	9.51E-08
DNAJC12	99.45687504	226.7704642	1.1908	1.22E-07

IL6	343.8656127	821.7088115	1.256	1.32E-07
NOCT	445.021942	1025.216967	1.2033	1.40E-07
MFSD2A	342.8920165	869.2398936	1.3406	1.41E-07
N4BP3	182.7967481	369.7073718	1.0133	1.51E-07
STARD7-AS1	94.21523439	210.9340194	1.1619	1.87E-07
C3orf80	40.78337383	152.5403331	1.898	1.90E-07
LAT2	48.67869249	144.7164897	1.5706	2.01E-07
L3MBTL2-AS1	5.877150883	62.10420079	3.3775	2.37E-07
LOC107986858	212.8544854	104.0220403	-1.031	2.65E-07
IL23A	19.84832218	78.96874266	1.9849	3.17E-07
OVGP1	85.06557943	187.6758099	1.1432	3.85E-07
LINC00623	105.8986185	214.3801332	1.0184	3.90E-07
NBR2	106.8317913	213.4445308	1.002	4.19E-07
TRIM6	190.5565092	65.8859902	-1.5295	4.94E-07
MON1A	138.2512015	315.3827379	1.1868	5.24E-07
DSEL	119.4196461	50.08495313	-1.2537	5.32E-07
ANGPT4	4.309543978	48.04067383	3.4612	6.51E-07
CRELD2	1775.867742	5117.227301	1.5268	9.80E-07
FAM174B	65.17480221	182.4264954	1.4801	1.14E-06
DSEL-2	127.224554	57.36147677	-1.1505	1.48E-06
MSC	102.1438002	249.9475861	1.289	1.57E-06
CGRRF1	87.41453693	175.4016877	1.0054	1.74E-06
RAB4B-EGLN2	149.7090357	62.22008601	-1.2661	1.84E-06
CHAC1	262.8059987	1666.380519	2.6641	3.09E-06
ZNF853	20.59547065	65.2457499	1.6608	3.46E-06
DNAJC3-DT	26.9100878	75.16039073	1.4853	3.56E-06
EREG	1378.018063	5491.954452	1.9946	4.33E-06
B3GNT4	249.150665	122.7506622	-1.0197	4.55E-06
SPRY4	379.7456107	811.5189933	1.0941	4.58E-06
LOC124904067	78.66964001	163.6402922	1.0537	5.66E-06
C2orf15	50.68657569	121.8812582	1.2671	6.79E-06
TAL1	48.74907182	107.5774122	1.1405	7.54E-06
OGA	4023.36129	1400.474777	-1.5224	9.48E-06
GMDS-DT	48.66667342	107.7620578	1.147	9.51E-06
LOC84214	8.74101136	39.21276771	2.1571	1.08E-05
GALNT4	177.5190491	83.51329821	-1.0912	1.24E-05
UGDH-AS1	51.91819058	110.7933162	1.0938	1.29E-05
FCGBP-2	25.18157026	75.11692882	1.5774	1.37E-05
SERTM2	81.24091904	26.73912093	-1.5972	1.48E-05
SLFNL1-AS1	91.38743112	258.3625212	1.4966	1.51E-05
LOC124901064	16.80525357	55.58716903	1.7197	1.60E-05
LOC124903025	1.683251891	27.08143719	4.0014	1.70E-05
SLN	104.5910042	41.98777185	-1.3099	1.72E-05
KLHL14	856.3104361	415.6630337	-1.0421	1.78E-05

ALPP	95.74092344	38.41762651	-1.3188	1.81E-05
LINC01224	86.34795944	34.59049089	-1.3158	2.36E-05
FGFBP1	44.51511005	10.74932582	-2.0558	2.36E-05
ZFYVE28	60.64144282	140.017204	1.2016	2.39E-05
VAC14-AS1	136.1836401	66.2860822	-1.0382	2.40E-05
LOC124904086	30.73115921	76.99227986	1.3222	2.68E-05
CPA4	163.4230244	80.92540945	-1.0115	2.81E-05
ANKRD1	327.9187846	702.1376579	1.0978	2.82E-05
LOC105372692	21.54706153	76.98511113	1.827	3.21E-05
KDM7A	61.23061068	127.2969077	1.0579	3.72E-05
LOC124903520	31.351302	83.17669225	1.409	3.94E-05
LOC730338	111.8277306	44.38490252	-1.3307	4.07E-05
LOC101928994	5.729993211	32.16255936	2.4838	4.25E-05
LOC100506207	58.63409679	126.1409766	1.1085	4.71E-05
BEX4	64.26100186	140.4696165	1.1295	6.28E-05
FAM241A	45.122696	113.5909884	1.3288	6.44E-05
TICAM2	148.8594568	73.91335492	-1.0115	6.54E-05
NMRK2	42.06240604	101.8126115	1.2688	7.23E-05
ADGRG2	288.8040742	890.1633042	1.6241	8.63E-05
ARX	5.140286985	28.32349867	2.4669	8.76E-05
EGR1	816.0339582	2068.377179	1.3417	9.27E-05
FCGBP	15.57429704	49.71690243	1.6719	0.000112
PLA2G4C	41.98054541	86.7288	1.047	0.000118
PPM1K	99.52564047	202.7892638	1.0294	0.000122
UGT1A9	9.760709304	0	-5.7671	0.00013
LOC101930053	32.4425754	81.43433369	1.3361	0.000133
PRKAR2A-AS1	29.76647373	71.08014919	1.2516	0.000139
DEPP1	103.2173139	42.70497379	-1.2709	0.000155
LOC107985005	12.78428429	40.80780644	1.6699	0.000163
APLF	36.52991785	84.71782608	1.214	0.000175
TMEM200C	2.965752492	23.4686918	2.9595	0.000176
LOC124900387	41.22245399	100.6890791	1.2837	0.000187
C10orf55	84.94365603	39.09318237	-1.1209	0.000201
ACSM5	91.74010614	43.81966098	-1.0618	0.000207
LOC124904012	28.25896158	68.64431294	1.2755	0.000218
LINC03011	40.44498896	86.33417666	1.0941	0.000249
NKX3-2	27.26222564	67.58235869	1.3058	0.00025
FGF21	0	9.270506057	5.632	0.000253
LOC124902640	26.36498887	65.15294507	1.308	0.000266
ANG	30.20902228	77.1776614	1.3502	0.000292
LOC124907917	0	9.135939386	5.607	0.000301
CAVIN4	73.63871959	151.8242659	1.0433	0.000307
SOWAHB	48.09580245	110.5196445	1.2017	0.000311
DLG1-AS1	8.57782846	34.89746795	2.0288	0.000319

MKX	0	9.031645482	5.5924	0.000335
SLFN12L	33.4925899	70.04541144	1.0635	0.000389
KRT81	23.54866631	62.15217739	1.3956	0.000413
IL10RA	13.97117129	40.8396534	1.5486	0.000423
TMEM217B	11.05642511	0	-5.9467	0.000427
GDPD3	50.83026497	20.87972319	-1.287	0.000437
ZNF790-AS1	42.4242901	86.63705137	1.0359	0.000443
LOC124903862	0	8.473882782	5.4984	0.000459
LOC124901263	1.683251891	18.74243119	3.4707	0.00049
LOC124902520	46.1498685	103.7201196	1.1683	0.00051
FAM27E3	15.41458254	42.08341495	1.4565	0.00054
LOC124905557	84.22921637	32.91025206	-1.3497	0.000563
LOC124902384	5.720246402	25.62665179	2.1545	0.000584
HSPA2	43.33970376	16.98551717	-1.3561	0.000606
SFMBT2	30.40892016	66.44258093	1.1308	0.000658
KBTBD8	40.29609739	85.70480522	1.0823	0.000686
TMOD2	13.22121279	47.47837279	1.8432	0.000741
ABCB11	7.567877325	0	-5.3953	0.000759
LRRC31	6.852779304	27.30658052	1.9983	0.000802
EGR3	8.971763562	35.8844471	1.988	0.000848
FAM43A	32.45058831	74.66527586	1.2017	0.000903
FAM167A-2	10.12313172	31.92907533	1.6528	0.000908
PCGF3-AS1	169.5068911	349.7411111	1.0423	0.00098
FOSB	105.1624141	431.9583779	2.0373	0.00099
RDH12	2.360021038	15.80165729	2.7341	0.001043
LOC100996437	41.80988796	89.34096475	1.0921	0.001113
MIR210HG-2	316.6542704	153.7782787	-1.0404	0.001219
SLC15A2	24.45672543	54.53364759	1.1561	0.00122
SGSM1	7.060569496	26.60550823	1.9079	0.001258
PINLYP	83.39715663	170.0028253	1.0265	0.001267
HOXD1	24.27351114	54.99196483	1.174	0.001273
SKIDA1	91.75104908	42.53265551	-1.1079	0.001283
ABLIM2	20.44831298	49.0293294	1.2644	0.001317
RND1	33.86595526	70.74617362	1.0673	0.001356
TROAP-AS1	5.409368061	24.1304383	2.1571	0.001358
AJUBA-DT	29.29815314	61.20263975	1.0579	0.001454
LOC124902456	15.57710707	44.72341642	1.5177	0.001454
PAPPA2	5.361292378	21.48515238	1.9928	0.001516
LOC105374174	9.677114774	30.38729764	1.6415	0.001574
LOC105370092	35.34703701	80.38461461	1.1909	0.001701
LOC124903753	1.314551057	12.54040708	3.2254	0.001908
RAB39B	3.735862979	24.40145593	2.7066	0.001971
PTGS2	3.690597323	18.07530814	2.28	0.001975
ATP6V1C2	30.42655929	72.77191087	1.259	0.00199

LOC105377113	0	6.585676352	5.1326	0.002031
LOC124902652	41.30658629	15.85470033	-1.391	0.002423
HID1	2.565001203	20.79430509	2.9827	0.002441
SAP25	68.33979363	34.11761819	-1.0051	0.002461
LHFPL4	0	6.183148309	5.044	0.002629
KLF4	446.4107038	1293.918695	1.535	0.002722
MIR210HG	62.48668089	28.02369034	-1.153	0.002898
C9orf43	15.52903139	38.63792139	1.3097	0.003054
LOC124903676	0	5.946079917	4.9846	0.003229
ANXA8	44.35312329	20.2077517	-1.137	0.0033
MYBPH	45.06248066	19.83759871	-1.1826	0.003402
RASGRP1	30.97058209	64.1011237	1.0571	0.003509
LINC01119	33.15647729	12.43283891	-1.4177	0.003638
LOC124903058	1.955801358	13.82982494	2.7853	0.003671
SLFNL1	38.48464368	101.8036404	1.3992	0.003679
LOC100505498	9.918151554	0.641388618	-3.935	0.003726
GRM8	5.277697848	22.50154243	2.0707	0.003917
C11orf65	8.436411437	24.55721869	1.5397	0.003997
RHBDL1	50.73064522	23.31367514	-1.1143	0.004043
LOC102724467	1.70274551	12.3117715	2.8518	0.004062
LEKR1	24.04275894	48.36548061	1.0075	0.004121
FAM167A	9.177281496	25.56696812	1.4809	0.004134
STEAP3-AS1	13.71064089	36.87890508	1.4185	0.004216
LOC124904771	4.736067304	18.93488935	1.9958	0.004252
PTHLH	17.4905734	54.99650317	1.6427	0.004266
PLA2G4B	3.049347022	80.57619987	4.724	0.004314
RBM14-RBM4	5.58630393	21.36735923	1.955	0.004504
NDUFA4L2	30.3109143	64.16660979	1.0769	0.004536
PROB1	27.29080771	55.33691149	1.0138	0.004597
MYADML2	1.734795965	12.21971277	2.8384	0.004666
LOC107984210	6.134212891	21.07419153	1.7889	0.004684
SULT1E1	22.96069398	64.11039473	1.4889	0.004716
ACTA1	5.425393288	0	-4.9183	0.004774
LOC105369724	18.76452332	43.09854092	1.1939	0.004862
SSX5	15.8668779	3.556428089	-2.1496	0.005122
LOC105372309	0.673300756	9.915479041	3.8755	0.005242
PPP1CB-DT	20.43174998	47.0039259	1.1945	0.005688
LMOD1	79.55593266	32.00658872	-1.3074	0.005825
SLC1A7	10.21300467	0.962082927	-3.3949	0.006017
LOC105371399	0.673300756	9.52138381	3.8114	0.006027
LOC124901730	17.7098443	40.28368288	1.1852	0.00617
LOC100507634	12.16880603	29.49354919	1.2757	0.006188
C1QL4	35.6390801	14.7511	-1.2626	0.006259
MPP7	30.21302844	60.79831952	1.0139	0.006426

AUTS2	0	5.192274691	4.7929	0.006466
LOC124909400	5.024641999	18.78503124	1.8901	0.006565
LOC107987091	9.784209082	0.607221378	-3.9378	0.006597
AHRR	53.68730926	14.12373874	-1.9176	0.00663
FUT1	3.206251504	79.8810568	4.6274	0.006793
LUARIS	33.9460814	14.0704777	-1.2716	0.006944
LOC124904309	20.99287415	47.99891199	1.1856	0.00762
FBXO16	0	4.936330496	4.7168	0.007659
MAP4K1	6.454838042	20.70202836	1.6788	0.007741
NKD1	0	4.869788199	4.7001	0.00788
DUSP5	1589.647153	3226.294464	1.0211	0.007912
LOC124904469	8.996877235	29.28020505	1.6909	0.007921
LOC124901611	0	7.223480604	5.2677	0.007985
LOC124901011	6.384458713	26.6651919	2.0564	0.008142
LOC105379395	23.06138987	48.04636049	1.055	0.008635
LY6K	81.82141681	38.30109743	-1.0982	0.008658
LOC101929577	0	5.018164231	4.7375	0.008666
LOC124903221-2	9.818531796	25.6248596	1.379	0.008744
LOC124903939	38.86709748	17.28554349	-1.1726	0.00894
LOC124904106	1.955801358	11.74557599	2.5525	0.009023
LOC107984311	8.97804198	25.19860731	1.4785	0.009209
LOC107985057	1.346601513	13.19866132	3.2813	0.009261
LOC401021	5.986517451	23.29278916	1.9481	0.009376
RBM26-AS1	18.38661345	38.41762651	1.0644	0.009394
FOXD4	12.56674729	31.81329235	1.3463	0.009477
MAP1LC3C	76.00047394	30.96087988	-1.2887	0.009767
TDRD10	22.11847027	48.12640204	1.1125	0.009809
PDE4C	10.99914038	29.39620602	1.4213	0.009815
LOC105371903	16.59399499	4.968705553	-1.7548	0.010047
ATP6V0D2	5.008616772	18.95883159	1.9133	0.010106
ICA1	17.67432545	39.34248593	1.1573	0.01016
PTGES3L	23.85954465	52.49264255	1.1328	0.010183
LOC107986583-2	0	196.5863229	10.035	0.010217
PDCD6-AHRR	31.92432463	68.10499005	1.0895	0.010298
BTBD16	13.89385518	3.237525963	-2.0995	0.010495
LOC124909470	0.961875451	9.584341741	3.2622	0.010608
CCL20	12.34454576	33.52800854	1.4333	0.010939
LOC107986583	0	179.1032548	9.9009	0.011278
DNAAF8	10.92529266	28.20897977	1.3728	0.011357
LRRC7	15.78328337	4.501427396	-1.7993	0.011768
CFAP53	17.91482446	36.81773933	1.0359	0.012105
LOC124902750	0	4.649803428	4.6291	0.012329
ZNF516-AS1	4.229955608	16.48407177	1.9883	0.012543
LOC105372401	38.9688689	18.97264096	-1.0429	0.013017

LOC124902235	7.551852097	22.48445881	1.5764	0.013134
SMKR1	8.285247605	23.49115197	1.4886	0.013287
SSX2	42.57198554	18.42890561	-1.2033	0.01343
OPRL1	15.21815305	33.37394585	1.135	0.01348
LOC105376675	4.347872852	0	-4.6018	0.013618
LOC124900936	15.57082865	33.81728176	1.1171	0.013938
LOC107985355	7.153910835	19.46565354	1.4475	0.01404
IPO11-LRRC70	17.10518897	2.208900739	-2.9416	0.014253
LOC105371573	0	4.230191764	4.4966	0.014437
LOC102724859	7.137885608	20.01297325	1.4854	0.014475
MYZAP	0.993925907	8.187355713	3.0174	0.014604
C14orf178	0	4.294941878	4.5152	0.014893
LINC00173	12.43908323	2.882664414	-2.0941	0.015037
LTB-4	6.124466082	18.40823763	1.5914	0.015153
CAND2	2.376046266	10.71695076	2.1738	0.015207
LOC124901795	4.078791776	0	-4.5061	0.015346
LOC124905002	0	4.180733087	4.4821	0.015378
LOC124902257	4.094817004	0	-4.5109	0.015793
LINC00664	5.5027094	0.303610689	-3.9732	0.01602
DNMBP-AS1	6.775463192	0.656680055	-3.3799	0.016087
LOC124903033	10.5949207	25.46298432	1.2682	0.01613
LOC124903221	9.414312116	23.67011088	1.3207	0.016147
SLPI	19.91242309	41.02630914	1.0397	0.01623
AGAP11	8.122064705	22.00009704	1.4327	0.016364
LOC105372165	5.403089642	16.74486441	1.6254	0.016688
LOC105373042	0.724844831	7.909479453	3.5164	0.016718
KMO	42.70311798	92.10739667	1.1105	0.016924
C1orf220	26.54766539	10.90993702	-1.2751	0.017096
ERVH48-1	4.162386306	0	-4.5313	0.017203
FOXO1	19.83857537	40.68337265	1.0358	0.017328
TNKS2-DT	12.39955823	28.30789713	1.1833	0.017843
LOC105375267	8.545778004	1.34752735	-2.6943	0.018091
WBP2NL	11.01396948	1.988915968	-2.4875	0.018135
CHKA-DT	16.77374089	36.83154869	1.1358	0.018222
GNGT2	21.10505074	8.056063302	-1.3891	0.018254
FAM27E4	16.89859491	33.95215854	1.0025	0.01829
SLC26A9-AS1	26.4302865	58.72914401	1.1422	0.018529
FAM220A	11.73600428	40.67400952	1.7942	0.018633
EDRF1-DT	14.22141711	31.25046321	1.1375	0.019141
LOC124904428	17.66176862	35.66857479	1.0154	0.02066
LOC105373058	29.55868354	64.41884374	1.1199	0.020689
OCLN-2	11.24244942	26.68048334	1.2451	0.020866
IL11	9.991461506	26.93463535	1.4195	0.02095
LOC124909360	7.028519041	20.19879078	1.5141	0.021076

LRRIQ4	4.325569206	14.8176423	1.7662	0.021198
TTLL13	8.215406044	20.85062239	1.3533	0.021554
LOC124901966	0	3.976039753	4.4036	0.021559
GREB1L-DT	3.758166625	0	-4.3869	0.021753
LOC124902488	14.47100457	33.14836653	1.1893	0.021988
HTR7	0	3.924788892	4.3872	0.022153
LOC107984018	3.783938663	0	-4.3952	0.022167
LRRC3-DT	3.783938663	0	-4.3952	0.022167
CARS1-AS1-2	1.087267246	8.60875956	3.0361	0.022213
LOC105378797	3.71636936	0	-4.3732	0.022273
LOC124904942	3.71636936	0	-4.3732	0.022273
TMC3-AS1	10.77412883	25.27864886	1.2269	0.022448
LOC124904632	5.797562513	17.43444763	1.5937	0.022572
LOC107985981	0	3.875330215	4.3715	0.023214
CPLANE1-AS1	3.79996389	0	-4.4006	0.023221
RRH	0.673300756	6.807453306	3.3303	0.023456
LOC101927604	9.844303834	22.8071633	1.2166	0.023866
PIK3C2G	3.72611617	0	-4.3763	0.024197
LOC124905030	22.2283746	9.437757892	-1.2345	0.024372
DLL4	7.173404454	18.75772263	1.3929	0.024438
LOC105374304	3.690597323	0	-4.3647	0.024684
GAPLINC	25.06819753	10.28025548	-1.2849	0.024854
BTG1-DT	0	3.861830961	4.3675	0.024893
TTLL10	5.152843821	0	-4.8425	0.025044
LOC728743	41.13778334	20.06802647	-1.0265	0.025075
RHOQ-AS1	4.425188963	14.11635201	1.6727	0.025096
NKD2	23.1564657	48.46798233	1.056	0.025188
LOC107985939	19.84766381	41.08716478	1.0468	0.025218
LOC105371754-2	16.96897424	6.202024112	-1.4554	0.025574
REELD1	3.706622551	0	-4.37	0.025704
NGEF	6.108440854	18.77458825	1.6126	0.025929
LINC02610	1.398145587	8.137897036	2.5657	0.025981
UTS2B	13.38493346	3.894206018	-1.7772	0.026159
INSC	4.6009287	18.76077889	2.0104	0.02624
KCNC1	7.820933173	1.34752735	-2.5673	0.026386
LTB-3	4.441214191	13.7021169	1.6292	0.027061
LTB-6	4.441214191	13.7021169	1.6292	0.027061
LTB-7	4.441214191	13.7021169	1.6292	0.027061
LOC107986997	13.52515435	27.53880046	1.0247	0.027068
GPHA2	0	3.605886766	4.2646	0.027389
LOC105369601	0.32062515	5.001080611	3.7703	0.027567
LOC105369601-2	0.32062515	5.001080611	3.7703	0.027567
CARS1-AS1	0	3.588803146	4.2587	0.027658
CT62	0	3.638261824	4.2756	0.027715

PSG8-AS1	0	3.638261824	4.2756	0.027715
LINC01881-2	4.752092532	13.96976816	1.556	0.028132
LINC01881-3	4.752092532	13.96976816	1.556	0.028132
ADAMTS14	4.823130226	0	-4.7464	0.028212
CYP26B1	23.31109792	47.23951222	1.0098	0.028578
DLGAP1	11.33232237	1.821664133	-2.5938	0.028607
LOC105373020	0	3.573511709	4.2536	0.028748
LOC112268295	5.755765248	15.95509976	1.4687	0.028841
LINC02041-2	0.336650378	4.839205325	3.731	0.029018
SSX2B	41.5522876	19.39098854	-1.0949	0.029348
RBMS3-AS3	19.54719064	8.122605599	-1.2698	0.029497
SLC10A5	12.74021477	27.43481667	1.1132	0.029856
ARIH2OS	13.86527312	28.70020017	1.0502	0.029925
EMILIN1	24.55742131	51.438811	1.0589	0.029941
LINC01719	0	3.670636881	4.2867	0.029949
ALOX5AP	19.19331891	45.54524602	1.2377	0.030041
FAM88B	7.44248553	19.43865503	1.3846	0.030246
LOC102724068	0.641250301	6.372550206	3.2639	0.030377
C10orf143	14.41718824	29.62125724	1.0445	0.030672
PDE6G	24.07827778	10.93186909	-1.1509	0.030857
LOC105372621	4.556859177	16.8590732	1.9007	0.031208
HOXC5	18.68493495	7.032814627	-1.3999	0.031422
LGALSL-DT	15.54852501	31.15460212	1.0008	0.031501
LOC107985948	3.379718982	0	-4.2361	0.031626
LOC124901984	3.379718982	0	-4.2361	0.031626
LINC00115	15.24045669	31.19773027	1.0325	0.031739
NALT1	25.11400096	11.15723041	-1.1758	0.032029
LOC124903543	0	3.541136652	4.2424	0.032257
ESPN	19.21388806	6.391426009	-1.5774	0.03253
LOC105369304	10.71403408	25.86372018	1.2788	0.03257
LTB	4.441214191	13.39850621	1.5962	0.032638
LTB-2	4.441214191	13.39850621	1.5962	0.032638
LTB-8	4.441214191	13.39850621	1.5962	0.032638
RPA4	6.691868662	17.97459861	1.4153	0.032762
NPHS1	7.201986519	19.40575187	1.4325	0.032794
AKR1D1	4.399416926	0.320694309	-3.6545	0.033113
OCLN	10.51760459	22.71488658	1.1112	0.033611
VSIG2	3.363693755	0	-4.2302	0.034466
H4C4	3.489085549	0	-4.2755	0.034889
HSD11B1-AS1	2.732190263	11.17758829	2.0416	0.035062
FOXA3	5.104768138	14.79190787	1.5331	0.035233
LOC101059948	4.508783493	0.320694309	-3.6838	0.035248
PITX3	30.11568095	15.01726919	-1.0075	0.035283
PKN2-AS1	0	4.547301707	4.6011	0.035904

OCLN-3	8.462183474	19.57807015	1.2071	0.036379
OR2B6	4.880952718	0.303610689	-3.798	0.036453
CYYR1	12.75223383	27.04273161	1.0816	0.03689
LOC124906126	20.59266063	8.613826002	-1.2491	0.036975
CT75	9.244850798	2.292526657	-2.015	0.036994
ARL14	3.385997401	0	-4.2385	0.037061
JPH3	5.361292378	16.41910366	1.6006	0.037315
CDH1	0	4.497843029	4.5866	0.03754
GALR2	5.762043667	15.48961379	1.4281	0.037563
LOC102723446	13.87155154	4.680386302	-1.5869	0.038223
NECTIN4-AS1	3.32189649	0	-4.2147	0.038489
GUCA1C	9.296394872	2.021291025	-2.2231	0.03854
LINC00626	8.464993501	1.552220684	-2.415	0.038703
KLHDC7B-DT	0	4.387218604	4.5542	0.038727
LOC101928977	0	3.2528174	4.1176	0.038837
LOC124902385	2.308476964	9.838493752	2.0687	0.039146
LOC105369576	0	3.302276078	4.1362	0.039454
DNAAF4-CCPG1	0	48.9102037	8.0284	0.039968
LOC124902931	21.04776602	9.265439615	-1.1943	0.040209
LOC101929770	0	3.268108837	4.1232	0.040276
C4orf51	0	4.335967744	4.5386	0.04054
HTR3A	4.28030355	14.88597678	1.7827	0.040805
MAFA	1.955801358	9.652676221	2.2709	0.040817
HCG20-3	0.32062515	4.213108144	3.5298	0.041191
CHRM5	0	3.26990102	4.1242	0.041237
LOC124904444	8.340797839	20.62198681	1.3166	0.041256
BTBD18	10.25827032	2.915039471	-1.8112	0.041296
CCDC87	15.03894492	29.98118523	1.0013	0.04137
PRDM12	4.687991621	13.68324109	1.5363	0.041524
LOC107984016	9.924429972	2.341985334	-2.1006	0.042433
WEE2-AS1	0	3.220442343	4.1054	0.042488
WEE2-AS1-2	0	3.220442343	4.1054	0.042488
LOC124904692	6.040871552	15.77793304	1.381	0.042527
NETO1	8.82460589	1.988915968	-2.1636	0.042657
AICDA	2.773987528	11.39609099	2.052	0.04323
ADGRG3	0	3.186275103	4.0921	0.043388
MAP4K1-2	5.729993211	15.16026867	1.3947	0.043474
CPAMD8	4.142892687	14.19154512	1.787	0.044919
LOC105371452	8.016166529	19.2232086	1.2519	0.045026
EFNB3	2.007345432	8.999270425	2.1578	0.045655
LOC124902029	1.651201435	7.917912265	2.2366	0.046506
LOC105372468	1.009951134	6.955829338	2.773	0.046529
LOC105373785	8.135279906	19.62573664	1.2682	0.04674
EPO	3.033321795	0	-4.0808	0.046841

LOC101928053	6.79148842	16.3869466	1.2752	0.04686
ANKRD31	4.739535696	15.64484845	1.7257	0.046954
C2orf50	3.126663134	0	-4.118	0.046969
P2RY1	12.16880603	4.312025499	-1.5126	0.047138
LINC02618	2.629102114	10.15918806	1.9262	0.047312
MAB21L2	3.017296567	0	-4.0743	0.047328
STX18-AS1	12.59251932	28.23480631	1.1615	0.047384
FOXD3-AS1	0	4.266151187	4.5082	0.047489
DNAH12	8.779340234	19.66348825	1.1574	0.048116
PEG3	11.27796827	24.47516696	1.1166	0.048129
N4BP2L2-IT2	10.36763689	2.915039471	-1.8238	0.048376
PRRT3-AS1	11.07526036	22.78501324	1.0337	0.048525
PLEKHG1	3.210257664	12.36629662	1.9729	0.048847
LOC124907753	38.0259493	11.70760638	-1.7128	0.048981
LOC105376353	12.5598105	4.163649467	-1.5845	0.049018
LINC02334	10.50157936	22.30370772	1.0826	0.049096
