# Supplementary material for: The Involvement of Endoplasmic Reticulum Stress during the Interaction between Calcium Oxalate Crystals and Renal Tubular Epithelial Cells
Source: Biology (Basel). 2024 Sep 27;13(10):774. doi: 10.3390/biology13100774 (PMC11504059; doi:10.3390/biology13100774)
Supplement: Supplementary file 1 [file biology-13-00774-s001.zip › Table S2.pdf]

Table S2. The detailed results of GO enrichment analysis.

Category	Description	Gene Ratio	Bg Ratio	pvalue	p.adjust	qvalue	geneID	Count
BP	response to endoplasmic reticulum stress	28/327	266/18870	2.23E-14	8.10E-11	7.11E-11	CTH/HERPUD1/ERN1/DNAJB9/NUPR1/SESN2/XBP1/CXCL8/DNAJC3/FICD/DDIT3/MANF/HYOU1/EDEM1/SYVN1/TRIB3/CEBPB/SDF2L1/HSPA5/ATF3/SELENOK/STC2/HSPA1A/PPP1R15A/DERL3/PDIA4/CHAC1/FGF21	28
BP	response to unfolded protein	20/327	137/18870	2.77E-13	5.03E-10	4.41E-10	CTH/HERPUD1/ERN1/DNAJB9/XBP1/DNAJC3/FICD/DDIT3/MANF/EDEM1/HSPA5/DNAJB5/ATF3/STC2/HSPA1A/PPP1R15A/DERL3/CHAC1/FGF21/HSPA2	20
BP	response to topologically incorrect protein	21/327	159/18870	5.23E-13	6.32E-10	5.55E-10	CTH/HERPUD1/ERN1/DNAJB9/XBP1/DNAJC3/FICD/DDIT3/MANF/EDEM1/SDF2L1/HSPA5/DNAJB5/ATF3/STC2/HSPA1A/PPP1R15A/DERL3/CHAC1/FGF21/HSPA2	21
BP	cellular response to unfolded protein	14/327	89/18870	3.95E-10	3.51E-07	3.08E-07	CTH/HERPUD1/ERN1/DNAJB9/XBP1/FICD/DDIT3/HSPA5/ATF3/STC2/HSPA1A/PPP1R15A/DERL3/FGF21	14
BP	endoplasmic reticulum unfolded protein response	13/327	75/18870	4.83E-10	3.51E-07	3.08E-07	CTH/HERPUD1/ERN1/DNAJB9/XBP1/FICD/DDIT3/HSPA5/ATF3/STC2/PPP1R15A/DERL3/FGF21	13
BP	cellular response to	15/327	109/18870	6.48E-10	3.92E-07	3.44E-07	CTH/HERPUD1/ERN1/DNAJB9/XBP1/FIC	15

	topologically incorrect protein						D/DDIT3/SDF2L1/HSPA5/ATF3/STC2/HSPA1A/PPP1R15A/DERL3/FGF21	
BP	intrinsic apoptotic signaling pathway in response to endoplasmic reticulum stress	12/327	65/18870	1.06E-09	5.50E-07	4.83E-07	HERPUD1/ERN1/XBP1/DDIT3/HYOU1/SYVN1/TRIB3/CEBPB/SELENOK/HSPA1A/PPP1R15A/CHAC1	12
BP	regulation of response to endoplasmic reticulum stress	13/327	82/18870	1.53E-09	6.93E-07	6.08E-07	HERPUD1/ERN1/DNAJB9/NUPR1/XBP1/FICD/DDIT3/MANF/HYOU1/SYVN1/HSPA5/HSPA1A/PPP1R15A	13
BP	negative regulation of response to endoplasmic reticulum stress	8/327	44/18870	7.69E-07	0.00031	0.000272	HERPUD1/DNAJB9/XBP1/HYOU1/SYVN1/HSPA5/HSPA1A/PPP1R15A	8
BP	amino acid import across plasma membrane	8/327	50/18870	2.13E-06	0.000772	0.000677	SLC3A2/SLC1A4/SLC7A1/SLC6A9/SLC7A11/SLC43A1/SLC7A5/PER2	8
BP	L-amino acid transport	10/327	90/18870	3.57E-06	0.001106	0.000971	SLC3A2/SLC1A4/SLC7A1/SLC6A9/SLC7A11/SLC43A1/SLC7A5/PER2/SLC1A7/KMO	10
BP	L-alpha-amino acid transmembrane transport	9/327	71/18870	3.66E-06	0.001106	0.000971	SLC3A2/SLC1A4/SLC7A1/SLC6A9/SLC7A11/SLC43A1/SLC7A5/PER2/SLC1A7	9
BP	response to starvation	15/327	218/18870	6.51E-06	0.001816	0.001594	SESN2/XBP1/PCK2/DDIT3/GPT2/CDKN1A/HSPA5/ATF3/ASNS/NUAK2/SLC7A5/MFSD2A/MAP1LC3C/FOXO1/FOXA3	15
BP	fat cell	16/327	248/18	7.30E-	0.00189	0.0016	XBP1/DDIT3/JDP2/T	16

	differentiation		870	06	1	59	RIB3/CEBPB/NR4A2/PER2/GDF6/IL6/NOCT/PTGS2/KLF4/SULT1E1/C1QL4/FOXO1/IL11	
BP	regulation of endoplasmic reticulum unfolded protein response	6/327	30/18870	1.08E-05	0.002616	0.002295	ERN1/DNAJB9/XBP1/FICD/HSPA5/PPP1R15A	6
BP	intrinsic apoptotic signaling pathway	18/327	319/18870	1.26E-05	0.002849	0.0025	HERPUD1/ERN1/NUPR1/XBP1/DDIT3/ARHGEF2/HYOU1/SYVN1/TRIB3/CEBPB/CDKN1A/SELENOK/HSPA1A/PPP1R15A/IER3/CHAC1/PTGS2/EPO	18
BP	chaperone cofactor-dependent protein refolding	6/327	32/18870	1.60E-05	0.003336	0.002927	SDF2L1/HSPA5/DNAJB5/HSPA1B/HSPA1A/HSPA2	6
BP	IRE1-mediated unfolded protein response	5/327	20/18870	1.90E-05	0.003336	0.002927	ERN1/DNAJB9/XBP1/FICD/HSPA5	5
BP	negative regulation of endoplasmic reticulum stress-induced intrinsic apoptotic signaling pathway	5/327	20/18870	1.90E-05	0.003336	0.002927	HERPUD1/XBP1/HYOU1/SYVN1/HSPA1A	5
BP	regulation of endoplasmic reticulum stress-induced	6/327	33/18870	1.93E-05	0.003336	0.002927	HERPUD1/XBP1/DDIT3/HYOU1/SYVN1/HSPA1A	6

	intrinsic apoptotic signaling pathway							
BP	response to nutrient levels	23/327	495/18870	1.93E-05	0.003336	0.002927	PSPH/SESN2/XBP1/PCK2/DDIT3/GPT2/CDKN1A/MAP1B/HSPA5/ATF3/ASNS/NUAK2/STC2/SLC7A5/GDF15/MFSD2A/FGF21/PTGS2/MAP1LC3C/FOXO1/CYP26B1/FOXA3/EPO	23
BP	positive regulation of transcription from RNA polymerase II promoter in response to endoplasmic reticulum stress	4/327	11/18870	2.65E-05	0.004378	0.003841	DDIT3/CEBPB/HSPA5/ATF3	4
BP	'de novo' post-translational protein folding	6/327	37/18870	3.82E-05	0.005786	0.005077	SDF2L1/HSPA5/DNAJB5/HSPA1B/HSPA1A/HSPA2	6
BP	sulfur amino acid transport	4/327	12/18870	3.93E-05	0.005786	0.005077	SLC3A2/SLC1A4/SLC7A11/SLC7A5	4
BP	response to lipopolysaccharide	18/327	348/18870	3.99E-05	0.005786	0.005077	XBP1/CXCL8/PCK2/CEBPB/CXCL2/CLDN1/SLC7A5/IL6/NOCT/TRIM6/ANKRD1/TICAM2/IL10RA/PTGS2/SLPI/KMO/AICDA/EPO	18
BP	regulation of fat cell differentiation	11/327	146/18870	4.90E-05	0.006835	0.005997	XBP1/DDIT3/JDP2/TRIB3/CEBPB/IL6/NOCT/PTGS2/SULT1E1/C1QL4/FOXO1	11
BP	neutral amino acid transport	7/327	58/18870	6.20E-05	0.008237	0.007228	SLC3A2/SLC1A4/SLC6A9/SLC7A11/SLC43A1/SLC7A5/SLC1A7	7

BP	amino acid transmembrane transport	9/327	101/18870	6.51E-05	0.008237	0.007228	SLC3A2/SLC1A4/SLC7A1/SLC6A9/SLC7A11/SLC43A1/SLC7A5/PER2/SLC1A7	9
BP	'de novo' protein folding	6/327	41/18870	6.97E-05	0.008237	0.007228	SDF2L1/HSPA5/DNAJB5/HSPA1B/HSPA1A/HSPA2	6
BP	cellular response to starvation	12/327	179/18870	7.03E-05	0.008237	0.007228	SESN2/XBP1/CDKN1A/HSPA5/ATF3/ASNS/NUAK2/SLC7A5/MFSD2A/MAP1LC3C/FOXO1/FOXA3	12
BP	monosaccharide biosynthetic process	9/327	102/18870	7.04E-05	0.008237	0.007228	SESN2/PCK2/SLC39A14/GPT2/ATF3/MST1/PER2/DSEL/FOXO1	9
BP	response to molecule of bacterial origin	18/327	369/18870	8.46E-05	0.009597	0.008421	XBP1/CXCL8/PCK2/CEBPB/CXCL2/CLDN1/SLC7A5/IL6/NOCT/TRIM6/ANKRD1/TICAM2/IL10RA/PTGS2/SLPI/KMO/AICDA/EPO	18
BP	cellular response to external stimulus	17/327	346/18870	0.000122	0.01339	0.011749	SESN2/XBP1/IRF1/CDKN1A/HSPA5/ATF3/ASNS/NUAK2/NR4A2/SLC7A5/GADD45A/MFSD2A/ANKRD1/PTGS2/MAP1LC3C/FOXO1/FOXA3	17
BP	protein folding	13/327	223/18870	0.000146	0.015614	0.0137	ERO1B/DNAJC3/HYOU1/SDF2L1/DNAJB11/HSPA5/DNAJB5/HSPA1B/HSPA1A/PDIA4/APLF/HSPA2/PTGES3L	13
BP	muscle organ development	17/327	354/18870	0.00016	0.016592	0.014558	NUPR1/XBP1/RCAN1/ATF3/MAFF/HBEGF/USP2/MSC/ANKRD1/EGR1/CAVIN4/MKX/EGR3/ACTA1/DLL4/CYP26B1/NPHS1	17
BP	gluconeogene	8/327	90/188	0.0001	0.01699	0.0149	SESN2/PCK2/SLC39	8

	sis		70	69	4	11	A14/GPT2/ATF3/MST1/PER2/FOXO1	
BP	response to peptide hormone	19/327	430/18870	0.000196	0.019219	0.016863	SESN2/XBP1/PCK2/SLC39A14/GPT2/GRB10/TRIB3/MAP1B/STC2/KLF15/NR4A2/AREG/GDF15/DDR2/EREG/EGR1/FGF21/PTGS2/FOXO1	19
BP	circadian regulation of gene expression	7/327	70/18870	0.000207	0.019622	0.017217	GFPT1/PER1/ID1/USP2/PER2/NOCT/EGR1	7
BP	hexose biosynthetic process	8/327	93/18870	0.000212	0.019622	0.017217	SESN2/PCK2/SLC39A14/GPT2/ATF3/MST1/PER2/FOXO1	8
BP	cellular response to glucose starvation	6/327	50/18870	0.000216	0.019622	0.017217	SESN2/XBP1/HSPA5/ASNS/NUAK2/SLC7A5	6
BP	positive regulation of transcription from RNA polymerase II promoter in response to stress	4/327	18/18870	0.000224	0.019789	0.017363	DDIT3/CEBPB/HSPA5/ATF3	4
BP	regulation of transcription from RNA polymerase II promoter in response to stress	5/327	33/18870	0.000241	0.020862	0.018305	DDIT3/CEBPB/HSPA5/ATF3/EGR1	5
BP	amino acid transport	10/327	150/18870	0.000293	0.024711	0.021682	SLC3A2/SLC1A4/SLC7A1/SLC6A9/SLC7A11/SLC43A1/SLC7A5/PER2/SLC1A7/KMO	10
BP	chaperone-mediated protein folding	7/327	75/18870	0.000318	0.026262	0.023044	SDF2L1/HSPA5/DNAJB5/HSPA1B/HSPA1A/PDIA4/HSPA2	7

BP	negative regulation of apoptotic signaling pathway	13/327	243/18870	0.000338	0.027172	0.023842	CTH/HERPUD1/XBP1/SLC35F6/ARHGEF2/HYOU1/SYVN1/HSPA1B/HSPA1A/NR4A2/IER3/PTGS2/EPO	13
BP	response to hyperoxia	4/327	20/18870	0.000344	0.027172	0.023842	CDKN1A/SLC7A5/FOXO1/EPO	4
BP	cellular response to chemical stress	15/327	317/18870	0.00045	0.034743	0.030485	ERN1/SESN2/DDIT3/ARHGEF2/SLC7A11/TREX1/HSPA1B/HSPA1A/NR4A2/ETV5/DDR2/IL6/PTGS2/FOXO1/EPO	15
BP	positive regulation of cytokine production	20/327	499/18870	0.000474	0.035233	0.030915	CEBPG/XBP1/DDIT3/ARHGEF2/IRF1/CEBPB/SELENOK/HSPA1B/HSPA1A/RAB7B/SLC7A5/IL6/IL23A/TRIM6/EREG/TICAM2/EGR1/PTGS2/RASGRP1/LTB	20
BP	maintenance of blood-brain barrier	5/327	38/18870	0.000476	0.035233	0.030915	CLDN1/IL6/MFSD2A/PTGS2/OCLN	5
BP	regulation of autophagy	16/327	355/18870	0.0005	0.036271	0.031826	ERN1/NUPR1/SESN2/RNF41/DDIT3/TRIB3/LAMP3/BMF/SLC7A5/TRIM6/DEPP1/IL10RA/RAB39B/ATP6V1C2/ATP6V0D2/FOXO1	16
BP	lymphocyte differentiation	18/327	429/18870	0.000531	0.03684	0.032325	CEBPG/DNAJB9/NFIL3/XBP1/TNFRSF9/RNF41/SYVN1/IRF1/IL6/IL23A/EGR1/EGR3/RASGRP1/IL11/DLL4/CYP26B1/AICDA/ADGRG3	18
BP	regulation of DNA-templated transcription in response to stress	5/327	39/18870	0.000538	0.03684	0.032325	DDIT3/CEBPB/HSPA5/ATF3/EGR1	5

BP	L-glutamate import	5/327	39/18870	0.000538	0.03684	0.032325	SLC1A4/SLC7A11/PER2/SLC1A7/KMO	5
BP	intrinsic apoptotic signaling pathway in response to osmotic stress	3/327	10/18870	0.000565	0.037299	0.032727	ARHGEF2/PTGS2/EPO	3
BP	leucine transport	3/327	10/18870	0.000565	0.037299	0.032727	SLC3A2/SLC43A1/SLC7A5	3
BP	regulation of apoptotic signaling pathway	17/327	398/18870	0.000618	0.040052	0.035143	CTH/HERPUD1/NUPR1/XBP1/SLC35F6/DDIT3/ARHGEF2/HYOU1/SYVN1/ATF3/HSPA1B/HSPA1A/NR4A2/IER3/PTGS2/LTB/EPO	17
BP	negative regulation of fat cell differentiation	6/327	61/18870	0.000644	0.040202	0.035274	DDIT3/JDP2/TRIB3/IL6/C1QL4/FOXO1	6
BP	skeletal muscle tissue development	10/327	166/18870	0.000652	0.040202	0.035274	NUPR1/RCAN1/ATF3/MAFF/MSC/ANKRD1/EGR1/ACTA1/CYP26B1/NPHS1	10
BP	response to oxidative stress	17/327	400/18870	0.000654	0.040202	0.035274	ERN1/SESN2/RCAN1/SLC7A11/SELENOK/TREX1/STC2/HSPA1B/HSPA1A/NR4A2/ETV5/AREG/DDR2/IL6/ABCB11/PTGS2/FOXO1	17
BP	ERAD pathway	8/327	111/18870	0.000702	0.041835	0.036708	HERPUD1/DNAJB9/XBP1/EDEM1/SYVN1/SDF2L1/HSPA5/DERL3	8
BP	positive regulation of wound healing	6/327	62/18870	0.000703	0.041835	0.036708	XBP1/CLDN1/HBEGF/DDR2/EMILIN1/OCLN	6
BP	integrated stress response signaling	5/327	42/18870	0.000762	0.043387	0.038069	CEBPG/DDIT3/CEBPB/HSPA5/PPP1R15A	5

BP	protein folding in endoplasmic reticulum	3/327	11/18870	0.000767	0.043387	0.038069	ERO1B/DNAJC3/HSPA5	3
BP	carboxylic acid biosynthetic process	15/327	334/18870	0.000771	0.043387	0.038069	CTH/PSPH/XBP1/PSAT1/TRIB3/ASNS/DHRS9/PER2/DSEL/ACSM5/ABCB11/PTGS2/KMO/ALOX5AP/AKR1D1	15
BP	mononuclear cell differentiation	19/327	481/18870	0.000777	0.043387	0.038069	CEBPG/DNAJB9/NFIL3/XBP1/TNFRSF9/RNF41/SYVN1/IRF1/CEBPB/IL6/IL23A/EGR1/EGR3/RASGRP1/IL11/DLL4/CYP26B1/AICDA/ADGRG3	19
BP	negative regulation of intrinsic apoptotic signaling pathway	8/327	114/18870	0.000837	0.04503	0.039511	HERPUD1/XBP1/ARHGEF2/HYOU1/SYVN1/HSPA1A/PTGS2/EPO	8
BP	liver development	9/327	142/18870	0.00084	0.04503	0.039511	CEBPG/XBP1/PCK2/CEBPB/CLDN1/HNF1B/SLC7A5/IL6/UGT1A9	9
BP	organic acid biosynthetic process	15/327	337/18870	0.000844	0.04503	0.039511	CTH/PSPH/XBP1/PSAT1/TRIB3/ASNS/DHRS9/PER2/DSEL/ACSM5/ABCB11/PTGS2/KMO/ALOX5AP/AKR1D1	15
BP	circadian rhythm	11/327	204/18870	0.000895	0.047088	0.041317	NFIL3/GFPT1/PER1/ID1/USP2/PER2/NOCT/EGR1/EGR3/OPRL1/HTR7	11
CC	endoplasmic reticulum chaperone complex	4/342	11/19886	2.58E-05	0.009024	0.008848	HYOU1/SDF2L1/DNAJB11/HSPA5	4
CC	apical part of cell	20/342	469/19886	0.000196	0.023408	0.02295	SLC3A2/SLC39A14/SLC7A1/SLC6A9/SLC7A11/SLC43A1/CL	20

							DN1/ANK2/SLC7A5/DDR2/PARD6A/ADGRG2/IL10RA/ABCB11/SLC15A2/PAPPA2/ATP6V0D2/INSC/OCLN/P2RY1	
CC	apical plasma membrane	18/342	403/19886	0.000232	0.023408	0.02295	SLC3A2/SLC39A14/SLC7A1/SLC6A9/SLC43A1/CLDN1/ANK2/SLC7A5/DDR2/PARD6A/ADGRG2/IL10RA/ABCB11/SLC15A2/PAPPA2/ATP6V0D2/OCLN/P2RY1	18
CC	aggresome	5/342	35/19886	0.00031	0.023408	0.02295	EDEM1/HSPA1B/HSPA1A/KLHL14/SFMBT2	5
CC	endoplasmic reticulum protein-containing complex	9/342	126/19886	0.000334	0.023408	0.02295	ERN1/PIGA/HYOU1/SYVN1/SDF2L1/DNAJB11/HSPA5/TREX1/DERL3	9
MF	misfolded protein binding	8/338	20/18496	1.19E-09	7.20E-07	6.41E-07	DNAJB9/DNAJC3/EDEM1/SDF2L1/DNAJB11/HSPA5/HSPA1A/DERL3	8
MF	neutral L-amino acid transmembrane transporter activity	7/338	46/18496	1.86E-05	0.005263	0.004687	SLC3A2/SLC1A4/SLC6A9/SLC7A11/SLC43A1/SLC7A5/SLC1A7	7
MF	L-amino acid transmembrane transporter activity	8/338	66/18496	2.61E-05	0.005263	0.004687	SLC3A2/SLC1A4/SLC7A1/SLC6A9/SLC7A11/SLC43A1/SLC7A5/SLC1A7	8
MF	unfolded protein binding	10/338	122/18496	8.20E-05	0.012384	0.011028	ERN1/ERO1B/HYOU1/SYVN1/DNAJB11/HSPA5/DNAJB5/HSPA1B/HSPA1A/HSPA2	10
MF	amino acid transmembrane transporter activity	8/338	85/18496	0.000162	0.015614	0.013906	SLC3A2/SLC1A4/SLC7A1/SLC6A9/SLC7A11/SLC43A1/SLC7A5/SLC1A7	8

MF	protein-folding chaperone binding	10/338	133/18496	0.000168	0.015614	0.013906	ERN1/DNAJB9/DNAJC3/FICD/SYVN1/SDF2L1/HSPA5/DNAJB5/HSPA2/PTGES3L	10
MF	growth factor activity	11/338	162/18496	0.000198	0.015614	0.013906	INHBE/MANF/NGF/HBEGF/AREG/GDF15/GDF6/IL6/EREG/FGF21/IL11	11
MF	carboxylic acid transmembrane transporter activity	11/338	164/18496	0.000221	0.015614	0.013906	SLC3A2/SLC1A4/SLC7A1/SLC6A9/SLC7A11/SLC43A1/SLC7A5/MFSD2A/ABCB11/SLC1A7/SLC10A5	11
MF	organic acid transmembrane transporter activity	11/338	165/18496	0.000233	0.015614	0.013906	SLC3A2/SLC1A4/SLC7A1/SLC6A9/SLC7A11/SLC43A1/SLC7A5/MFSD2A/ABCB11/SLC1A7/SLC10A5	11
MF	transmembrane receptor protein tyrosine kinase activator activity	4/338	18/18496	0.000274	0.016533	0.014724	HBEGF/AREG/ANGPT4/EREG	4
MF	organic anion transmembrane transporter activity	13/338	242/18496	0.000536	0.026569	0.023661	SLC3A2/SLC1A4/SLC39A14/SLC7A1/SLC6A9/SLC7A11/SLC43A1/SLC33A1/SLC7A5/MFSD2A/ABCB11/SLC1A7/SLC10A5	13
MF	protein tyrosine/threonine phosphatase activity	3/338	10/18496	0.00066	0.026569	0.023661	DUSP16/DUSP8/DUSP5	3
MF	aromatic amino acid transmembrane transporter activity	3/338	10/18496	0.00066	0.026569	0.023661	SLC3A2/SLC7A1/SLC7A5	3
MF	branched-chain amino	3/338	10/18496	0.00066	0.026569	0.023661	SLC3A2/SLC43A1/SLC7A5	3

	acid transmembrane transporter activity							
MF	MAP kinase tyrosine phosphatase activity	3/338	10/18496	0.00066	0.026569	0.023661	DUSP16/DUSP8/DUSP5	3
MF	ATP-dependent protein folding chaperone	5/338	40/18496	0.00077	0.029068	0.025887	HYOU1/HSPA5/HSPA1B/HSPA1A/HSPA2	5
MF	histone chaperone activity	3/338	12/18496	0.001177	0.041827	0.037249	JDP2/APLF/PRDM12	3
MF	protein folding chaperone	6/338	66/18496	0.001288	0.043209	0.03848	HYOU1/HSPA5/HSPA1B/HSPA1A/APLF/HSPA2	6
MF	cytokine activity	12/338	237/18496	0.001445	0.045596	0.040606	INHBE/CXCL8/CXCL2/AREG/GDF15/GDF6/IL6/IL23A/CCL20/IL11/LTB/EPO	12
MF	MAP kinase tyrosine/serine/threonine phosphatase activity	3/338	13/18496	0.00151	0.045596	0.040606	DUSP16/DUSP8/DUSP5	3
